# Repression of Connexin26 hemichannel activity protects the barrier function of respiratory airway epithelial cells against LPS-induced alteration

**DOI:** 10.1186/s12964-025-02228-6

**Published:** 2025-05-16

**Authors:** Tina Lehrich, Anne Dierks, Masina Plenge, Helena Obernolte, Klaudia Grieger, Katherina Sewald, Frederic Rodriguez, Lucie Malet, Peter Braubach, Florence Bedos-Belval, Anaclet Ngezahayo

**Affiliations:** 1 Institute of Cell Biology and Biophysics, Department of Cell Physiology and Biophysics, Leibniz University Hannover, Hannover, Germany; 2 Fraunhofer Institute for Toxicology and Experimental Medicine (ITEM), Division of Preclinical Pharmacology and Toxicology, Hannover, Germany; 3 Biomedical Research in Endstage and Obstructive Lung Disease Hannover (BREATH), German Centre for Lung Research, Hannover Medical School, Hannover, Germany; 4 Laboratoire de Synthèse et Physicochimie des Molécules d’Intérêt Biologique, CNRS, UMR 5068, Toulouse, France; 5 Institute for Pathology, Hannover Medical School, Hannover, Germany; 6 Center for Systems Neuroscience (ZNS), University of Veterinary Medicine Hannover Foundation, Hanover, Germany

**Keywords:** Connexin channels; lipopolysaccharide, Airway epithelium, Cytokine, Barrier function, Primary cells, PCLS, Calu-3 cells

## Abstract

In respiratory airway epithelial cells, lipopolysaccharide (LPS) treatment induced an enhancement of connexin 26 (Cx26) hemichannel activity shown by dye uptake experiments after siRNA-mediated knock-down of Cx26. This effect was already observed at infection relevant concentrations (≤ 10 ng/mL LPS) and involved tumor necrosis factor alpha (TNF-α)- and Ca^2+^-dependent signaling. High concentrations (1 µg/mL LPS) reduced the transepithelial electrical resistance (TEER) of Calu-3 cells by 35% within an application time of 3 h followed by a recovery. Parallel to barrier alteration, a reduced tight junction organization rate (TiJOR) of claudin-4 (CLDN4) by 75% was observed within an application time of 3 h. After TEER recovery, CLDN4 TiJOR stayed reduced. Low concentrations (10 ng/mL LPS) required three times repeated application for barrier reduction and CLDN4 TiJOR reduction by 30%. The small molecule CVB4-57, newly published as a potential inhibitor of Cx26 hemichannels, mitigated the effects of LPS on the epithelial barrier function. Molecular docking studies revealed a potential interaction between CVB4-57 and Cx26 thereby reducing its hemichannel activity. We conclude that LPS-related enhancement of Cx26 hemichannel activity acts like a “molecular scar” that weakens the lung epithelium, which could be attenuated by agents targeting Cx26 hemichannels.

**Supplementary Information:**

The online version contains supplementary material available at 10.1186/s12964-025-02228-6.

## Introduction

In mammals, inflammation is triggered by both extrinsic and intrinsic signals [1, 2]. Extrinsic signals include invaders and their released pathogen-associated molecular patterns (PAMPs) which activate the pattern recognition receptors (PRRs) such as toll-like receptors (TLRs). Intrinsic signals, also known as injury signals, consist of cytokines, reactive oxygen species (ROS) and intracellular metabolites such as nucleotides that are released into tissues. These damage-associated molecular patterns (DAMPs) are released by the tissue’s own cells in response to physical, chemical or biological insults that damage cells or compromise the integrity and the permeability of the cell membrane This disruption leads to an uncontrolled release of intracellular metabolites into the tissue interstitium [3]. DAMPs in turn bind to their specific receptors on various cells and may contribute to the progression of the inflammatory response [4].

Epithelial tissue separates the external environment from the organism. Epithelial cells are therefore the first cells to be confronted by invaders and their PAMPs. Moreover, epithelial cells are highly susceptible to attack by both physical and chemical agents. As a result, they play a key role in responding to invaders and their PAMPs, while also contributing to the release of DAMPs [4, 5].

The receptors for PAMPs and DAMPs, known as pattern recognition receptors (PRRs), are expressed in both epithelial cells [5] and in the innate immune cells [2]. Due to the relative ease of isolating innate immune cells from peripheral blood, investigations into receptors and their physiological signaling pathways have mainly been conducted in leukocytes, in particular neutrophils and macrophages [2]. Since invaders and their released PAMPs have to cross the epithelial barrier to interact with blood cells, understanding the cellular responses of epithelial cells to PAMPs could help to develop a better management of inflammatory reactions. These reactions primarily occur when epithelial cells are stimulated by invaders and their released PAMPs, and subsequently by self-released DAMPs.

In this context, an enhancement of the activity of connexin (Cx) hemichannels in the epithelial cells in response to PAMPs such as lipopolysaccharide (LPS) and DAMPs such as adenosine has been shown [6, 7]. Cxs are membrane proteins found in almost all vertebrate tissues. In humans, they are encoded by a family of 21 genes [8] and are designated by the abbreviation “Cx” (for connexin) followed by a numerical suffix representing the protein’s approximate molecular weight in kilodaltons (kDa). For example, Cx26 refers to a connexin with a molecular weight of 26 kDa. The expression of Cx isoforms is regulated according to the development and the metabolic and physiological state of a tissue [10, 11]. Like other membrane proteins, Cxs are synthesized and inserted into the membrane of the endoplasmic reticulum (ER). Despite differences in the length of the C-terminal chain, all Cx isoforms adopt a similar membrane topology: four transmembrane domains (TM1-4) linked by two extracellular loops and one cytoplasmatic loop with the N- and C-termini both located on the cytoplasmatic side. Depending on the isoform, the Cx polypeptides oligomerize in the ER or in the ER-Golgi intermediate compartment and the Golgi network [12]. They form hexamers called connexons, which are exported to the cell membrane, where they may stay as hemichannels or form cell-to-cell gap junction channels between adjacent cells. The opening of Cx hemichannels would allow diffusion of ions and small metabolites (up to 1.2–1.5 kDa) between the extracellular and the intracellular space along the electrochemical gradient of the particular ions or metabolites. The mechanisms that control the activity of Cx hemichannels are not fully understood. However, it is recognized that an external Ca^2+^ concentration ([Ca^2+^]_ex_) of around 2 mM strongly reduces the opening probability of single Cx hemichannels [13]. In addition, the docking of the Cx hemichannels to form Cx gap junction channels contributes to the maintenance of a low density of Cx hemichannels in cell membrane [14]. Recently, results show that the activity of Cx hemichannels is upregulated under inflammatory conditions in various tissues such as skeletal muscles (which normally do not express Cxs), respiratory airways, the intestine and brain [15–17]. The mechanistic relationship between increased Cx hemichannel activity and its role in pathologies or inflammation is the subject of ongoing research. An upregulation in Cx expression, particularly for Cx43 and Cx26, has been widely observed under inflammatory conditions [6, 15, 18]. The use of specific Cx hemichannel inhibitors, such as antibodies or mimetic peptides (Gap19, primarily targeting Cx43), have reported changes in the regulation of expression, the turnover or the phosphorylation state [10]. Recent results reveal that other Cx isoforms like Cx26 may also be involved [6, 15, 18]. Furthermore, the individual biological properties of each Cx isoform are the subject of ongoing research [9]. This suggests that with respect to function, regulation and involvement in processes at the cellular and tissue-levels, consideration should be given to each isoform individually rather than an extrapolation of findings on Cx43 alone [10–18].

Airway epithelial cells express Cx26, Cx30, Cx31, Cx32, Cx37, Cx43 and Cx45 at varying levels [19–22]. In these cells, Cx channels are involved in various tissue functions such as coordinated cilia beat or secretion of surfactants [19, 20, 23]. It is assumed that Cx hemichannels in the epithelial cells can be opened due to their mechanical sensitivity [19, 23]. The contribution of this normal opening of the Cx hemichannels to the tissue function is understood. To date, changes of Cx hemichannel activity in the context of inflammation in the pulmonary tissue have not been systematically investigated. Nevertheless, it has already been observed that LPS, one of the prominent PAMPs of gram-negative bacteria [24], induced an upregulation of Cx43 expression in the kidneys and the lungs of rats [25]. The DAMP adenosine has recently been shown to increase the activity of Cx26 hemichannels in model cells of the respiratory airways [15, 26]. Respiratory epithelial cells contribute to LPS-induced pulmonary inflammation in vivo by releasing cytokines such as tumor necrosis factor alpha (TNF-α) and interleukin-1 [27]. The physiological significance of an increased Cx hemichannel activity is matter of ongoing research. It can be assumed that a prolonged increased Cx hemichannel activity may lead to a long-lasting release of injury signals in tissue, which may continue to further stimulate inflammatory reactions even after the decay of infection. As a result, the tissue functions such as the barrier function of the epithelial tissue may be affected [18]. In this context, an increased Cx hemichannel activity may represent a detrimental response, which in turn is involved in the establishment of long-lasting chronic inflammation in epithelial tissue following infection or primary injuries.

The present study demonstrates that LPS induces an increase in Cx26 hemichannel activity through a signaling dependent on TLR4 activation and TNF-α secretion. This was investigated using Calu-3 cells as a model for the respiratory airways along with human primary bronchial epithelial precursor cells (PBEPCs) expanded from donor lung tissue [28]. Additionally, we found that LPS raised the presence of Cx26 in the epithelial layer of human precision cut lung slices (PCLS). Finally, LPS affects the integrity of tight junctions in Calu-3 cells cultivated on transwell inserts. Tight junctions are multi-protein structures of the cell-to-cell contact regions that form a gasket seal of the apical region of the epithelial cells. They control the cell polarity and restrict the paracellular flux of ions and metabolites [29]. Among these proteins, claudins (CLDNs) appear to be the major structural and functional components of the tight junctions [30]. CLDNs are a family of membrane tetraspan proteins with 27 isoforms in mammals. To form a tight seal between cells, the CLDNs are anchored on the cytoskeleton via scaffolding proteins like proteins of the zonula occludens (ZO) family [31, 32]. In cell culture, tight junctions can be morphologically visualized by staining CLDNs or ZOs and functionally investigated by measuring the transepithelial electrical resistance (TEER). Using these methods with Calu-3 cells, we observed a reduction of the TEER values and a remodeling of the tight junction proteins after LPS-treatment. Furthermore, we found that LPS also affects the presence of CLDNs in the epithelial and subepithelial layer of PCLS.

In the context of inflammation, development of drugs targeting Cx hemichannels for treatment of chronic inflammation and the maintenance of tissue function has been proposed [33]. In this regard, the phenolic compound CVB4-57 [26] has recently been shown to specifically target Cx26 hemichannels. The specificity may be due to mutual interactions. Latest structural studies carried out on Cxs show that lipid-like molecules interact within the Cx hemichannels, at the neighboring of N-terminal helices (NTH), either formally (ligand structures are resolved) or hypothetically (structures are not resolved). The channel could be partially filled with lipids in open, intermediate or closed states. This is the case for Cx43 hemichannels in closed state (steroid, phospholipids) [34], for Cx36 hemichannels in open state (phospholipids) [35] and in the structure of Cx26 hemichannels putative lipid-like densities are found [36, 37]. In Cx31.3 hemichannels, a lipid binding groove [38] was identified. Docking studies have positioned potential Cx channel inhibitors within these regions, as seen with Cx46 channels (carbenoxolone, enoxolone) [39] and Cx43 channels (pilocarpine) [40]. Various studies suggest that conformational equilibria may be based on different states [34, 35], depending on the experimental conditions (pH, lipid and detergent environment, CO_2_ partial pressure). These states are mainly described by the position of NTH, the α-to-π helix transition in the TM1 helix, eventually a specific reordering of residues in the pore [41] and the pore aperture. In this context, interacting compounds within the channel (and in particular at the interfaces between NTH and TM1 helices) could play a role in the structural dynamics of the channel. In the case of Cx43 channels [34, 35], a derivative of cholesterol and phospholipids, or unresolved lipids (pore lipid sites) [42] could be involved in stabilizing the closed state. These structural considerations applied to Cx26 hemichannels allowed to identify two specific hydrophobic pockets as putative binding sites for CVB4-57, which may explain its ability to reduce the dye uptake of LPS-treated cells to control levels and to alleviate the detrimental action of LPS on the barrier function. Based on these findings, we hypothesize that an enhanced activity of Cx hemichannels in the epithelial cells during an inflammatory response is a relevant mechanism involved in PAMP-induced weakening of the epithelia. Furthermore, it may be possible to restore the barrier function of epithelial cells under inflammatory conditions by targeting Cx26 hemichannels.

## Materials and methods

### Materials

LPS O26:B6 from *Escherichia coli*, C34, BAY-1797, BAPTA-AM were purchased from Sigma-Aldrich (Taufkirchen, Germany). Recombinant human TNF-α was from Bachem AG (Bubendorf, Switzerland). Marimastat, SDP-304 was from Biomol (Hamburg, Germany). All inhibitors were preincubated for 30 min prior to addition of LPS or TNF-α. The diarylheptanoid (CVB4-57) was synthesized as previously described [26]. The vehicles H_2_O and DMSO were added to control cells in all experiments at maximal concentrations of 0.5% or 0.1%, respectively.

### Cell culture

The human primary bronchial epithelial precursor cells (PBEPCs) were isolated from tissues obtained during tumor resections or lung transplantation with full consent of the patients (Ethics approval: ethics committee Hannover Medical School, no. 2699–2015). Tissue material from donor trachea or explanted lung was stored up to 16 h in RPMI supplemented with penicillin/streptomycin and amphotericin B at 4 °C. The method for the generation of PBEPC cultures were used as described early [43]. The cells were cultivated in modified keratinocyte medium (Gibco, Thermo Fisher Scientific, Waltham, MA, USA) and were used up to passage 4. The human lung adenocarcinoma epithelial Calu-3 cells (AddexBio, San Diego, CA, USA, Cat #: C00116001, LOT 0179286) were cultured in Dulbecco’s MEM/Ham’s F-12 medium (Biochrom, Berlin, Germany) supplemented with 10% fetal calf serum (Biochrom, Berlin, Germany), 1 mg/mL penicillin, and 0.1 mg/mL streptomycin (Biochrom, Berlin, Germany). The cells were maintained in a cell culture incubator in a humidified atmosphere with 5% CO_2_ at 37 °C. The cell culture medium was renewed every three days. Cells up to passage 35 were used for experiments.

### Knockdown of Cx isoforms

For siRNA-mediated knockdown of Cx isoforms in Calu-3 cells, 30 × 10^5^ cells/cm^2^ were seeded on collagen I-coated coverslips and grown for 24 h to a confluence of about 30%. Cell culture media was changed to Opti-MEM (Thermo Fisher Scientific). Cx26-siRNA (Qiagen, Hilden, Germany, SI03047856 and SI03084809), Cx43-siRNA (SI00003493 and SI02780491) and Silencer Select Negative Control No. 2 siRNA (Thermo Fisher Scientific) were diluted in JetPrime dilution buffer (Polyplus transfection, Illkirch, France) to a final siRNA concentration of 9.6 nM per 48-well and 26.4 nM per 24-well. Per 48-well 1 µL, per 24-well 1.5 µL JetPrime transfection reagent (Polyplus transfection) were added. The transfection mix was incubated for 15 min at room temperature before addition to the cells. After 6 h the transfection medium was replaced by pre-warmed standard cell culture medium and cells were cultivated for 48 h before dye uptake experiments, or quantification of mRNA amount was performed as well as protein amount detection by western blot experiments.

### Dye uptake assay

**Fig. 1:**
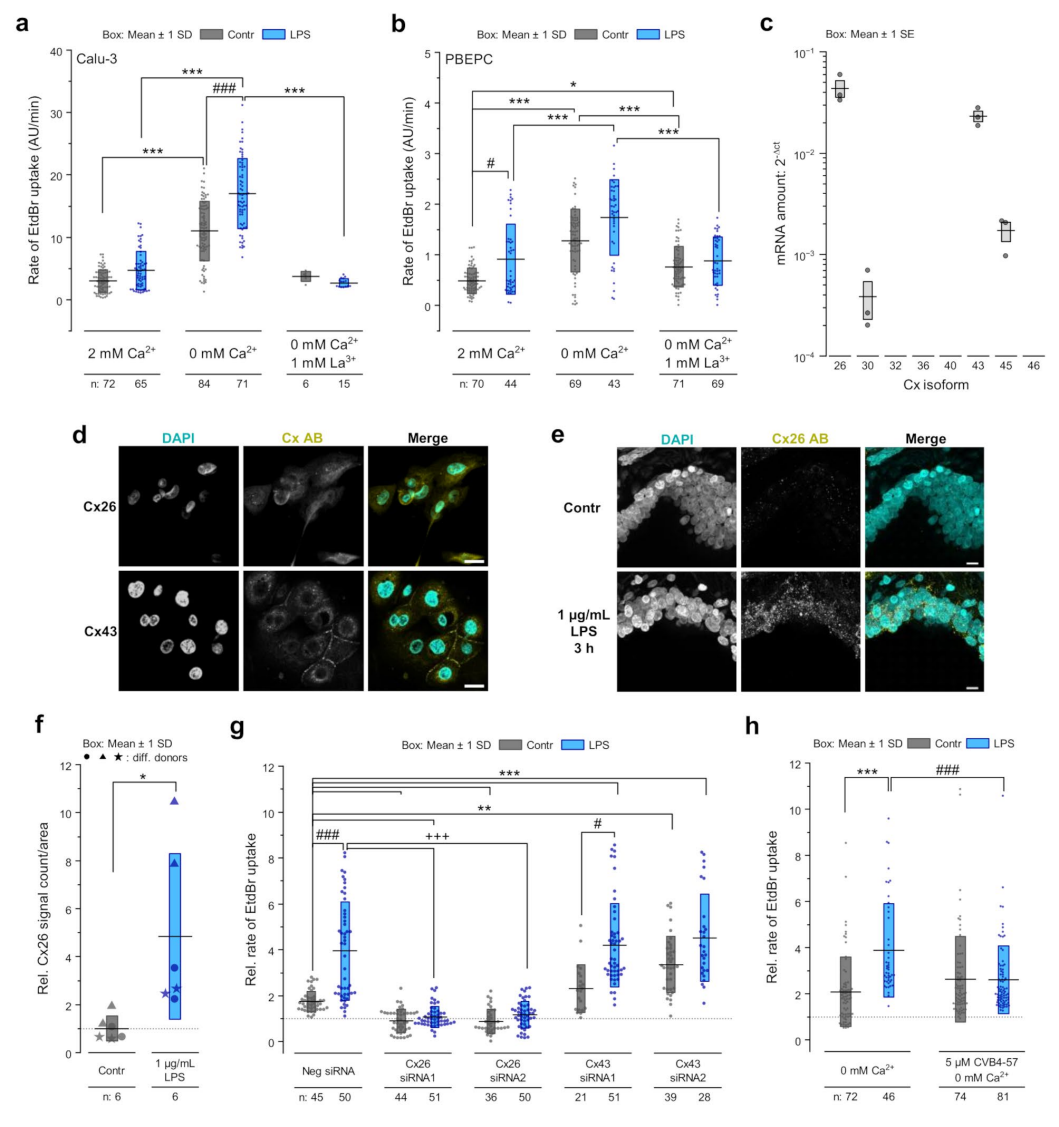
LPS enhances the EtdBr dye uptake in airway epithelial cells. a-b EtdBr dye uptake rates (fluorescence intensities (AU)/min) under different perfusion conditions (exemplary experiment see Fig. S1) in Calu-3 cells (**a**) and PBEPCs (**b**) cultivated under control conditions or with 1 ng/mL LPS for 24 h. Perfusion conditions: 2 mM [Ca^2+^]_ex_, 0 mM [Ca^2+^]_ex_ or 0 mM [Ca^2+^]_ex_ + 1 mM La^3+^ (n = cell patches for Calu-3 cells, single cells for PBPECs). Kruskal-Wallis test with Dunn’s multiple comparison test (*p* < 0.05 *, *p* < 0.001 *** vs. different perfusion conditions; *p* < 0.05 #, *p* < 0.001 ### vs. control). **c** Real-time qRT-PCR for different Cx isoforms in PBEPCs. **d** Immunofluorescence staining against Cx26 or Cx43 (yellow) in PBEPCs. Scale bar = 20 μm. **e** Exemplary immunofluorescence staining against Cx26 (yellow) in the airways of PCLS cultivated under control conditions or with 1 µg/mL LPS for 3 h. Scale bar = 10 μm. **f** Cx26 immunofluorescence signal (particle count/area, relative to control) in PCLS after treatment with 1 µg/mL LPS for 3 h (n = analyzed PCLS from 3 donors, 2 PCLS/donor with mean of 3 airway areas/PCLS). Different symbol shapes visualize different donors. Unpaired two-tailed Student’s *t*-test (*p* < 0.05 * vs. control). **g** EtdBr dye uptake rates in absence of [Ca^2+^]_ex_ relative to the rates obtained in presence of [Ca^2+^]_ex_ in Calu-3 cells in which Cx26 or Cx43 expression was suppressed using respective siRNA and which were treated with 1 ng/mL LPS or vehicle for 24 h (n = measured cell patches). Kruskal-Wallis test with Dunn’s multiple comparison test (*p* < 0.01 **, *p* < 0.001 *** vs. negative siRNA + vehicle; *p* < 0.05 #, *p* < 0.001 ### vs. respective siRNA + vehicle; *p* < 0.001 +++ vs. LPS). **h** EtdBr dye uptake rates in absence of [Ca^2+^]_ex_ ± 5 µM CVB4-57 relative to the rates obtained in presence of [Ca^2+^]_ex_ in Calu-3 cells treated with vehicle or 1 ng/mL LPS for 24 h (n = cell patches). Kruskal-Wallis test with Dunn’s multiple comparison test (*p* < 0.001 *** vs. vehicle; *p* < 0.001 ### vs. LPS)

The activity of Cx hemichannels was analyzed by measuring the ethidium bromide (EtdBr) uptake as described previously [15, 44]. Calu-3 cells as well as PBEPCs were cultivated on collagen I-coated coverslips (diameter 5 mm) to 40% confluence. With this, the Calu-3 cells had grown to cell patches in the size of 500-4,000 µm^2^, whereas on cell covered approximatively 100 µm^2^. PBEPCs remained mainly as single cells. The cells were placed in a perfusion chamber with a volume of approximately 400 µl and mounted on an Eclipse Ti microscope (Nikon). Regions of interest (ROIs) were selected in a transmission micrograph of single cells for PBEPCs and cell patches for Calu-3 cells acquired with an Orca flash 4.0 CCD camera (Hamamatsu Photonics Deutschland GmbH, Herrsching am Ammersee, Germany). During the experiment, fluorescent images were taken every 15 s with an exposure time of 900 ms, 20x objective (except for data in Fig. 1b: 40x objective) and a wavelength of 560 nm, to assess fluorescence intensity changes within the ROIs using the NIS-Elements AR 4.4 software (Nikon GmbH). The ISMATEC REGIO ICC peristaltic pump (Cole-Parmer GmbH, Wertheim, Germany) was used to maintain a constant 500 µL/min medium flow rate. During the first 5 min of a 15 min long dye uptake experiment, the cells in the chamber were perfused with a pre-warmed (37 °C) bath solution containing 121 mM NaCl, 5.4 mM KCl, 6 mM NaHCO_3_, 5.5 mM glucose, 0.8 mM MgCl_2_, 1.8 mM CaCl_2_, 25 mM HEPES (pH 7.4, 295 mOsmol/L) and 5 µM EtdBr. For the next 5 min, the medium was changed to a Ca^2+^/Mg^2+^-free bath solution containing 1 mM EGTA. For some measurements 1 mM La^3+^ or 5 µM CVB4-57 was added to this solution. The dye uptake rate (AU/min) was calculated based on the slope of the linear regression of fluorescence intensity values within the first minute of the respective perfusion steps (Fig. S1). Further experiments with Calu-3 cells were normalized (rel. rate of dye uptake) by dividing the rate of the dye uptake from the Ca^2+^-free bath solution to the rate of the dye uptake from the Ca^2+^ containing bath solution from the same cells. For the results ≥ 3 biological replicas were used, and each measurement was performed with 2–4 coverslips per treatment group.

### Real time qRT-PCR

For real time qRT-PCR the PeqGOLD Total RNA kit (Peqlab, VWR International GmbH, Darmstadt, Germany) was used for total RNA isolation of cells grown on tissue culture plates according to the manufacturer’s protocols. The Maxima First Strand cDNA synthesis kit for qRT-PCR with dsDNase (Thermo Fisher Scientific) was used for cDNA synthesis. The primer pairs for gene expression analysis of Cx and CLDN isoforms were described previously [14, 64], claudin isoform 1 (CLDN1), toll like receptor subtype 4 (TLR4), tumor necrosis factor alpha (TNF- α), zonula occludens-1 (ZO-1) are given in Table 1. Real time qRT-PCR was used to quantify gene expression and gene expression changes after LPS treatment and to analyze RNA knockdown after siRNA transfection. The ΔΔct-method was used for quantification of the relative mRNA amounts using β-actin as housekeeping gene for normalization. The real time qRT-PCR was performed with the KAPA SYBR™ FAST Universal mastermix (Kapa Biosystems) in the Bio-Rad CFX384 cycler (Bio Rad). The PCR program included an initial denaturation at 95 °C for 3 min, 40 cycles of denaturation at 95 °C for 15 s, annealing of primers and elongation at 60 °C for 30 s, followed by melt curve generation. For the results, 3 biological replicas were used.

**Table 1:** List of primer pairs used for quantitative real time PCR not published previously [15, 45]

Target gene	Primer sequence 5’– 3’	Amplicon size [bp]
CLDN1	CCAGTCAATGCCAGGTACGA CAAAGTAGGGCACCTCCCAG	89
TLR4	CTGCAATGGATCAAGGACCA TTATCTGAAGGTGTTGCACATTCC	74
TNF-α	TCAGCCTCTTCTCCTTCCTG GCCAGAGGGCTGATTAGAGA	124
ZO-1	TGGTGTCCTACCTAATTCAACTCA CGCCAGCTACAAATATTCCAACA	134

### Immunofluorescence staining

For immunofluorescence staining, 10 × 10^4^ cells were seeded on collagen I-coated coverslips (diameter 5 mm) and grown for 24–48 h to a confluence of 70%. Additionally, the transwell inserts from the TEER measurement were used after measurements. The cells were fixed with an acetone/methanol mix (1:2) for 5 min at -20 °C or with 4% paraformaldehyde for 20 min at 4°C and blocked with 1% BSA with or without 0.01% TX-100 in phosphate-buffered saline (PBS) for 30 min at 37 °C. The primary antibodies anti-Cx26 antibody (4 µg/mL, Alomone Labs; ACC-212), anti-Cx43 antibody (0.75 µg/mL, Sigma-Aldrich, C6219), anti-TLR4 antibody (0.5 µg/mL, Thermo Fisher Scientific, 48-2300), anti-CLDN1 (5 µg/mL, Thermo Fisher Scientific, 51-9000), anti-CLDN3 (3 µg/mL, Thermo Fisher Scientific, 34-1700), anti-CLDN4 (2 µg/mL, Thermo Fisher Scientific, 32-9400) and anti-ZO-1 (1.25 µg/mL, Thermo Fisher Scientific, 40-2200) were diluted in PBS and added to the cells overnight at 4°C. The secondary iFluor488™-conjugated anti-rabbit and anti-mouse antibodies (AAT Bioquest, 16608, 16528) were diluted 1:1,000 in PBS with 2 µM DAPI (Sigma-Aldrich) and incubated for 1 h at 37 °C. The cells were washed with PBS and stored at 4°C. The immunostaining was imaged in z-stacks with an Eclipse TE2000-E inverse confocal laser-scanning microscope (Nikon GmbH) with a 60x water immersion objective and the software EZ-C1 (Nikon GmbH). For the figure preparation, ImageJ was used. First, the contrast was enhanced to 0.01% saturated pixels on all slices (normalized, used stack histogram), then the z-stack of the DAPI staining was combined in an average projection whereas the antibody signals were combined in a maximum projection, respectively. Image panels were created using the ImageJ plugin QuickFigures [46].

### PCLS experiments

Human precision-cut lung slices (PCLS) were prepared as described before [47]. Briefly, human lung lobes were cannulated and selected segments were inflated with 37°C warm 2% low-gelling agarose (Fisher Scientific, Schwerte, Germany) in Dulbecco’s modified Eagle’s medium nutrient Mixture F-12 Ham (pH 7.2–7.4) with L-glutamine and 15 mM HEPES (DMEM). The lobe was kept on ice until the agarose was polymerized and cut into 1.5-3 cm-thick slabs. Tissue cores with a diameter of 8 mm were cut with a rotating sharpened coring tool and sliced with a semiautomated microtome (Krumdieck tissue slicer, Alabama Research & Development, Munford, AL, USA) into about 250–350 µm-thick sections. The preparation was performed in Earle’s balanced salt solution. PCLS were subsequently washed three times with DMEM/F-12 with glutamine and HEPES supplemented with 100 U/mL penicillin/100 µg/mL streptomycin and cultivated under normal cell culture conditions (37°C, 5% CO_2_, 100% air humidity). One day after preparation, PCLS were stimulated with 1 µg/mL LPS for 3 h or 24 h. As control, vehicle was used. Afterwards, PCLS were fixed overnight and stored in PBS for further staining. Immunofluorescence staining was performed as described in Wronski et al. [48]. Shortly, PCLS were stained overnight at 4°C with primary antibodies rabbit anti-Cx26 antibody (Proteintech #16960-1-AP) and mouse anti-CLDN4 antibody (Thermo Fisher Scientific #32-9400). After washing for 6 h, secondary antibodies were incubated overnight at 4°C using donkey anti-mouse Cy3 and donkey anti-rabbit Cy5. PCLS were washed for 6 h and nuclei were stained using DAPI. After embedding in ibidi mounting medium images of PCLS were taken using confocal laser-scanning microscopy. Quantitative analysis of Cx26 expression was performed using ImageJ (version 1.8.0_345). The airway region was labelled in the DAPI channel and then transferred to the Cy5 channel for analysis. In the Cy5 channel, Cx26 particles were quantified using the “analyze particles” tool with the following settings: threshold range from 0-240, particle size from 10 to infinity (in inch², using pixel units), and circularity between 0–1”.

### Molecular graphics and structural analysis

Molecular graphics were performed with the UCSF Chimera package. Chimera is developed by the Resource for Biocomputing, Visualization, and Informatics at the University of California, San Francisco (supported by the NIGMS P41-GM103311). The protein structures used in this paper were downloaded from the RCSB Protein Database [34, 36–38, 42, 49–52] and split using Discovery Studio Visualizer (DSV 2021) from Dassault Systèmes Biovia (www.3dsbiovia.com). If the PDB entry existed in hexamer form (connexon, hemichannel) the structures were split to retain the chains A and B. If the structure existed as a dodecamer (channel), two chain from each hexamer were included in structural study (i.e. A, B and G, H chains) [53]. Cx43, Cx26, Cx36, Cx31.3 and Cx46/50 types found in the PDB were collected and compared. The resulting protein structures were aligned in the same reference space. Two Cx26 types were used as reference for structural alignments: the historical structure 2ZW3-4 [68] (chains AB, formerly 2ZW3ab or 2ZW3:AB), 5ER7-5 [54] for calcium atom positioning (chains AB) and the more recent entries 7QEV, 7QEW [36] (chains AB). The structure used for docking experiments was prepared (structure checks, rotamers, hydrogenation, splitting of chains) using DSV. The new compounds were sketched using ChemAxon Marvin 16 (www.chemaxon.com). CVB4-57 as potential binding compound was checked with respect to hybridization, hydrogenation, some geometry optimizations, 3D sketching and merged in SDF libraries using DSV.

### Molecular Docking

Molecular modelling studies were performed with Molegro Virtual Docker 6 (www.molexus.io) software using 7QEV (chains AB, formerly 7QEV: AB) PDB structure [35] in the reference space. The docking process occurred at the interface between NTHs and NTH-TM1 helices, which is well structured (far from the region of non-structured loops at the cytoplasmic side).

We completed a structural analysis (Cx43, Cx26, Cx36, Cx31.3 and Cx46/50 types) and cross correlation analysis of literature data, considering the position of crystallographic ligands after alignments, searching for residues close to hypothetical densities (i.e. so called ‘sausage shaped’ lipid densities), which could generally be used as docking sites. These collected data were then positioned on Cx26 structures such as 7QEW and 7QEV. The envelope and topology of these putative sites are used to direct ligand design. The molecular docking protocol was performed using two target sites (site-01 and site-02) close to NTH helixes of 7QEV: AB structure (ESI figure Z1). MSE/MolDock evaluator (simplex evolution algorithm) was used two internal scoring schemes (Moldock and Rerank) [55, 56] were combined in a consensus (scoring) approach. The protocol gave two sets of poses (one for each docking site). Non-displaceable water molecules were taken in account inner the binding sites. Clustering of poses (Tabu clustering) was set with an RMSD threshold of 2 Å. The docking process used 6000 iteration steps, and a grid resolution of 0.3 Å, along 20 independent runs. Internal parameters (population size, number of iterations, energy threshold.) of the algorithm were left as default. A final minimization (per run) was parameterized using 2000 steps for lateral chains and 2000 steps for protein backbone preceded by a minimization and optimization (hydrogen bonds) of the ligand (CVB4-57). The convergence was attained before the limit of iterations steps. Conformational space was correctly sampled, and all the poses were dispersed along the two potential sites. The reproducibility test gave the same results (pathways and fluctuation of poses) for each site.

Poses analysis (classification and manual grouping of poses) was carried out using scores (Moldock, Rerank) [55] and topology analysis. If a given pose was found to combine best values for the two scores, it was defined as strong. In consensus approaches, the strong poses usually correlated with lower RSMD values (deviation from crystallographic conformations). In this work, conformity criteria (i.e. comparison with liganded structures, similar chemical compounds, a core set of interactions) was missing. Therefore, the strong poses were clustered using their trajectories (pathways) in the cavities. We specifically focused on whether these poses shared a common conformation within the same group (i.e. engaged in hydrogen bonding or π-π interactions) and showed fluctuations in other regions of the molecule (i.e. aliphatic chains).

### Ca^2+^-Imaging

Changes in baseline intracellular Ca^2+^ concentrations ([Ca^2+^]_i_) were estimated using ratiometric Ca^2+^ imaging measurements with Fura-2 [57]. Calu-3 cells (6 × 10^4^) were cultivated on collagen I-coated coverslips (diameter 5 mm) and grown in cell patches to a confluence of 40%. Before the measurement cells were treated with 1 ng/mL LPS for 24 h, with 10 ng/mL TNF-α for 1 h or as vehicle control with 0.2% H_2_O with 0.1% BSA for 24 h. Cells were then incubated with 2 µM Fura-2-AM (Merck Millipore, Darmstadt, Germany) for 20 min at 37 °C in the dark followed by placement in a perfusion chamber on an inverted microscope Axiovert 10 (Carl Zeiss AG, Oberkochen, Germany) equipped with a 40x objective. Cells were washed under perfusion to remove external Fura-2 using the ISMATEC REGIO ICC peristaltic pump (Cole-Parmer GmbH) with a constant medium flow rate of 500 µL/min for 5 min with prewarmed (37 °C) bath solution containing 121 mM NaCl, 5.4 mM KCl, 6 mM NaHCO_3_, 5.5 mM glucose, 0.8 mM MgCl_2_, 1.8 mM CaCl_2_, 25 mM HEPES (pH 7.4, 295 mOsmol/L). The following measurement was performed under the same perfusion conditions for a total time of 800 s. It was controlled using an EPC 10 USB Double amplifier (HEKA, Multi-Channel Systems MCS GmbH, Stuttgart, Germany) and the software Patchmaster with the imaging software extension SmartLUX (HEKA, Multi-Channel Systems MCS GmbH). Fura-2 was excited every second at 360 and 380 nm with an exposure time of 7 ms using a Polychrom IV (T.I.L.L. Photonics GmbH) and emission was detected at 510 nm with a Retiga ELECTRO CCD camera (Teledyne Photometrics, Tucson, AZ, USA). Per measurement, up to 18 regions of interest (ROIs) were selected as single cells within a cell patch and the mean fluorescence intensity ratio F_360 nm_/F_380 nm_ was calculated for each second of the measurement. The average F_360 nm_/F_380 nm_ was formed from five minutes measurement. Cells with spontaneous changes in fluorescence intensities were not taken into account. Any measurement disturbances and artefacts were detected based on fluorescence intensity profiles and removed for the calculation of the average value. For the results ≥ 3 biological replicas were used, and each measurement was performed with 2–4 coverslips per treatment group.

### Western blot

Protein isolation after 48 h siRNA transfection was performed as described previously [14] 20 µg Protein were used for the SDS-PAGE run. Western blot was performed with primary antibodies anti-β-tubulin antibody as loading control (1:2,000, Sigma-Aldrich, T4026), anti-Cx26 antibody (0.5 µg/mL, Merck, MABT198) and anti-Cx43 antibody (0.19 µg/mL, Sigma-Aldrich, C6219), which were diluted in PBS-Tween and applied to the blotted nitrocellulose membranes at 4 °C overnight. The secondary horseradish peroxidase-coupled anti-rabbit and anti-mouse antibodies (1:40,000, Sigma-Aldrich, A9169 and A9044) were each applied for 1 h at room temperature. The detection was carried out with a substrate containing coumaric and linoleic acid (100 mM Tris pH 8.5, 1.25 mM linoleic acid, 0.225 mM coumaric acid, freshly added 0.01% H_2_O_2_) and imaged with a CCD camera imaging system (Intas Science Imaging, Göttingen, Germany). For the results, 3 biological replicas were used.

### Transepithelial electrical resistance (TEER) measurement and analysis of the tight junction organization rate (TiJOR)

For the TEER measurements, 10^5^ Calu-3 cells were seeded in transwell inserts (0.3 cm^2^) with a transparent PET membrane (pore size 0.4 μm, BD Falcon, Corning) and cultivated in cell culture medium for 3 days before being transferred into the cellZscope (nanoAnalytics, Muenster, Germany) and placed in the cell culture incubator. After 5 days, a stable barrier was formed by the cells (> 800 Ωcm^2^). The cells were then treated with 1 ng/mL– 1000 ng/mL LPS for 3.5–24 h. The TEER was monitored every 0.5 h by impedance spectroscopy, the data were automatically recorded by the cellZscope software. For the relative TEER values of treated cells, the measurement was first normalized to the starting TEER value and secondly to the cells cultivated under control conditions.

After the TEER measurement, the LPS treated cells at 3.5 h and 24 h were used for the analysis of the tight junction organization rate (TiJOR). Therefore, the stack images of immunofluorescence staining with ZO-1 and CLDN4 taken with the Eclipse TE2000-E inverse confocal laser scanning microscope (Nikon GmbH) were used with the macro “TiJOR parameter quantification for tight junction” (TiJORP) from Terryn et al.. 2013 [58]. The ImageJ software was used to convert stack immunofluorescence images into binary images. The central, best TJ structure-representing slide ± two slides were used for the zprojection tool. The image was binarized with subtract background and auto threshold. With the TiJOR macro (polygon iteration = 10, step width = 33 px) the number of intersections of TJ stricter to a mask of concentrically arranged rectangles with increasing perimeter was determined. The TiJOR was calculated as the mean of numbers of intersections normalized to rectangle perimeter and is given as intersections per µm (intersections/µm). For the results of the TEER measurement and the TiJOR estimation, at least three biological replicates were used, with each measurement performed using 1–4 transwell inserts per treatment group.

### Statistics

Statistical analysis was performed using OriginPro 2022b software (OriginLab Corporation, Northampton, MA, USA) and GraphPad Prism 8.0.4 (GraphPad Software Inc, Boston, MA, USA). First, data was checked for normal distribution via Shapiro-Wilk algorithm. Normal distributed data of two groups were compared using unpaired two-tailed Student’s *t*-tests, whereas multiple groups were compared using one-way analysis of variance (ANOVA) following Sidak’s, Tukey’s or Dunnet’s multiple comparisons tests. When normal distribution was rejected, nonparametric tests were performed. For this, the Kruskal-Wallis test was used for comparison of multiple groups followed by Dunn’s multiple comparisons tests. Levels of significance were indicated as * *p* < 0.05, ** *p* < 0.01 and *** *p* < 0.001. Further details of the statistical tests used for the respective results are given in the figure legends.

## Results

### Enhancement of Cx hemichannel activity in respiratory epithelial cells in response to LPS treatment

We analyzed the activity of Cx hemichannels in human PBEPCs expanded from donor lung tissue [28] and in Calu-3 cells. This was done by monitoring the uptake of the membrane-impermeable fluorescence dye ethidium bromide (EtdBr) into the cells in the presence or absence of [Ca^2+^]_ex_. The presence of [Ca^2+^]_ex_ maintained a low fluorescence intensity in the cells. The removal of [Ca^2+^]_ex_ correlated with a fast increase in fluorescence intensity in the first 3–4 min followed by a saturation (Fig. S1, 1a, 1b). The removal of [Ca^2+^]_ex_ did not increase the fluorescence intensity in HeLa cells [14] that do not express Cxs. Moreover, La^3+^, a classical inhibitor of Cx hemichannels antagonized the increase of fluorescence intensity after [Ca^2+^]_ex_ removal (Fig. 1a, b). These results suggest that the removal of [Ca^2+^]_ex_ triggered the opening of Cx hemichannels in both PBEPCs and Calu-3 cells, facilitating dye uptake into the cells as already shown for Calu-3 cells [15].

We used the dye uptake rate in the presence or absence of [Ca^2+^]_ex_ (Fig. S1) to compare control cells with cells treated for 24 h with 1 ng/mL LPS. Both PBEPCs and Calu-3 cells treated with LPS showed a higher rate of dye uptake in the presence of [Ca^2+^]_ex_ compared to cells cultivated under control conditions. This effect was even more pronounced following the removal of [Ca^2+^]_ex_. Adding La^3+^ as Cx hemichannel inhibitor prevented increased dye uptake after [Ca^2+^]_ex_ removal in both control and LPS treated cells (Fig. 1a, b). Using the Calu-3 cells, the pharmacology for the LPS-induced increase of the dye uptake rate was further analyzed revealing time- and concentration-dependency (Fig. S2a, b). A significant effect on the dye uptake rate after treatment for 24 h was first observed with 1 ng/mL LPS. The dye uptake rate steadily increased with rising LPS concentrations reaching a maximum of nearly double the rate of dye uptake compared to control conditions at a concentration of 3 ng/mL. Further increase of the LPS concentration did not correlate with a more enhanced rate of dye uptake (Fig. S2a). Regarding the time-dependency, a significant effect was observed after 3 h, with the magnitude depending on the LPS concentration applied. Prolonged application times up to 24 h did not further increase the observed effect (Fig. S2b). Incidentally, the increased dye uptake rate was unrelated to any change in cell viability caused by LPS treatment (Fig. S2c).

Quantitative real-time reverse transcriptase polymerase chain reaction (qRT-PCR) revealed that PBEPCs express low levels of the Cx isoforms Cx30 and Cx45 while Cx26 and Cx43 are expressed at higher levels (Fig. 1c). The latter was further confirmed by immunocytochemistry showing a diffuse distribution of Cx26 throughout the cell whereas Cx43 was clearly located at cell-cell-contacts (Fig. 1d). Additionally, immunostaining revealed a significant increased presence of Cx26 in the airways of precision cut lung slices (PCLS) treated with 1 µg/mL LPS for 3 h (Fig. 1e, f). We have recently shown that the activity of hemichannels in Calu-3 cells was mainly related to Cx26 [15]. Accordingly, siRNA-based repression of Cx26 expression suppressed the LPS-induced enhancement of the dye uptake rate in Calu-3 cells (Fig. 1g, S3). Of note, siRNA-based repression of Cx43, the most expressed Cx isoform in the cells, did not reduce the dye uptake rate (Fig. 1g). These results confirm our previous work [15, 26] and highlight the importance of Cx26 hemichannels in the dye uptake of Calu-3 cells. They also reveal an LPS-related enhancement of Cx26 hemichannel activity in the epithelial cells of the respiratory airways. Consistent with our recent findings, we observed that the small molecule CVB4-57, a diarylheptanoid and potential inhibitor of Cx26 hemichannels [26], effectively suppressed the enhanced dye uptake rate following [Ca^2+^]_ex_ removal in LPS-treated cells to control levels (Fig. 1h).

**Fig. 2:**
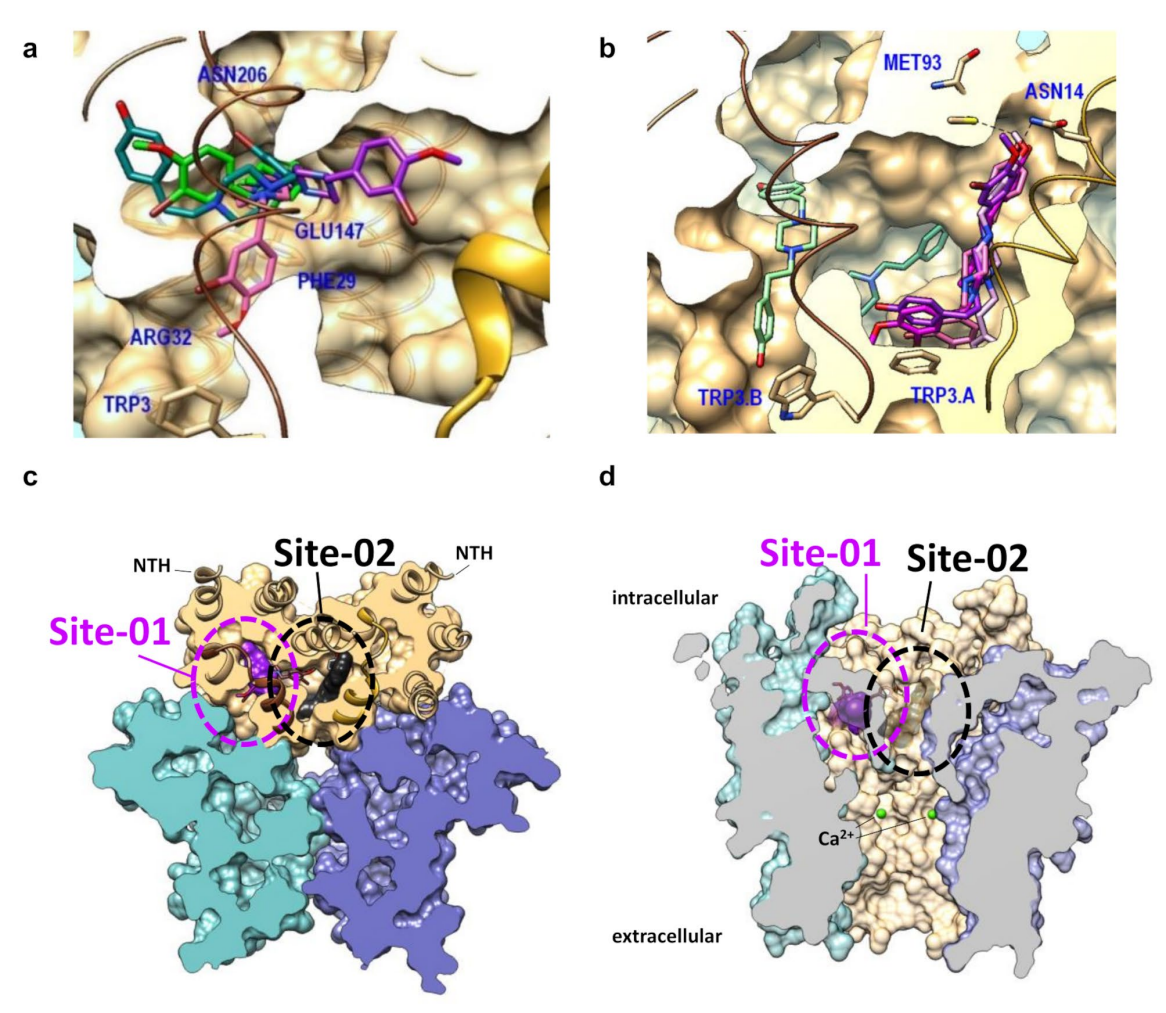
Molecular docking of CVB4-57 in the structure of Cx26 hemichannels. **a-b** Putative binding modes of CVB4-57 on the Cx26 7QEV structure (clipped molecular surface) based on the distribution of best docked poses in site-01 (**a**) and site-02 (**b**). NTHs, relative to site-01 cavity (left NTH in brown) and site-02 cavity (between NTHs) are shown in brown and gold (tubes, ribbons), respectively. Some tagged residues (navy blue) are shown below the molecular surface (transparency). For the molecular docking of CVB4-57 in site-01 (**a**), three pathways could be recorded: linear along the cavity (pink pose); constrained (green poses); extended towards site-02 cavity (purple). For the molecular docking in site-02 (**b**), a main linear fluctuation was recorded near the NTH (right, pink-purple). Poses were found in the central sub-cavity (teal) and in site-01 (light green). **c-d** Since the Cx26 structure 7QEV consists of only two chains (**a**, **b**, tan color), three 7QEV structures (tan, teal, blue) were aligned on 7QEW [35] (Cx26 type, hexamer) to reconstruct a hexamer. **c** Clipped hexamer (orthogonal, view from cytoplasmic side) showing best docked poses in site-01 (left, under brown ribbons) and site-02 (right, near gold ribbons). One linear pose of CVB4-57 per site was highlighted by molecular surface (site-01: purple, site-02: dark grey). **d** Laterally clipped hexamer showing the pore and molecular surfaces (transparency) of highlighted docked poses. The position of Ca^2+^ atoms (green spheres) is also shown after alignment with 5ER7 [54] (Cx26 type, dodecamer split as hexamer)

In silico approaches were utilized to predict putative binding pockets of CVB4-57 in Cx26 hemichannels, thereby giving a theoretical basis to understand how the small molecule may affect the Cx26 hemichannels. Firstly, a structural analysis (using Cx43, Cx26, Cx36, Cx31.3 and Cx46/50 types) and a cross-correlation analysis of the literature data were carried out [36, 37, 42, 51, 52, 54, 59]. The collected data was then positioned on Cx26 structures such as 7QEW and 7QEV (PDB databank registration) [60], allowing the identification of two putative sites (Fig. 2a, b) that could be used for molecular docking simulations. The first identified pocket (site-01) was located under the NTH of 7QEV (Fig. 2a, b) and the second one (site-02) between the NTHs of chains A and B. The site-01 includes 50 flexible residues whereas site-02 contains 49 flexible residues (some residues overlapping between the two sites). Secondly, CVB4-57 was subjected to a protein-ligand binding simulation process at both sites and exhibited promising binding poses (Fig. 2a, b). For site-01 (Fig. 2a, S4), three various groups of poses (linear along the cavity, curved-constrained, extended towards site-02) were recorded with a common origin in a cavity under the NTH. Phenol, bromo or methoxy groups of CVB4-57 were able to establish hydrogen interactions with residues ARG32, GLU147 and ASN206, surrounding the cavity. No specificity was observed for the CVB4-57 ligand orientation in site-01. Additional stacking (amide-π, π-π) interactions could be observed with LEU25 and PHE29 and eventually ARG143 (π-cation). For site-02 (Fig. 2b, S5), the main fluctuation concerned an alignment of poses in which aromatic cycles of CVB4-57 were involved in stacking interactions with TRP3. These interactions appear to be predominant and direct binding modes. Once again, no preference for the CVB4-57 ligand orientation was recorded. ASN14 and MET93 residues are located near the phenolic and bromo-methoxyphenolic moieties of CVB4-57, potentially contributing to its interaction. A sub-cavity in the center of site-02 was also occupied by poses, although the ligands efficiency scores are less interesting. In the two cases, the piperidine core provides flexibility inside the hydrophobic environment without major interactions. Figure 2c-d display these results in the context of a Cx26 hexamer, showing that linear poses could be assimilated to compounds occupying pore lipid sites. From a structural point of view (based on a static snapshot), these results clearly suggest that CVB4-57 could be able to bind in the cavity below the NTH of Cx26. The binding modes are not only sterically directed, but they also highlight some intriguing interactions. However, at this stage these modeling data represent a hypothetical prediction of how CVB4-57 may interact with Cx26 hemichannels and therefore inhibit their activity. Future experiments may corroborate or refute these findings, thereby offering a better understanding of the operational mechanisms of Cx26 hemichannels.

### TLR4 receptor, TNF-α and Ca^2+^ in the LPS-related enhancement of the Cx26-hemichannel activity

**Fig. 3:**
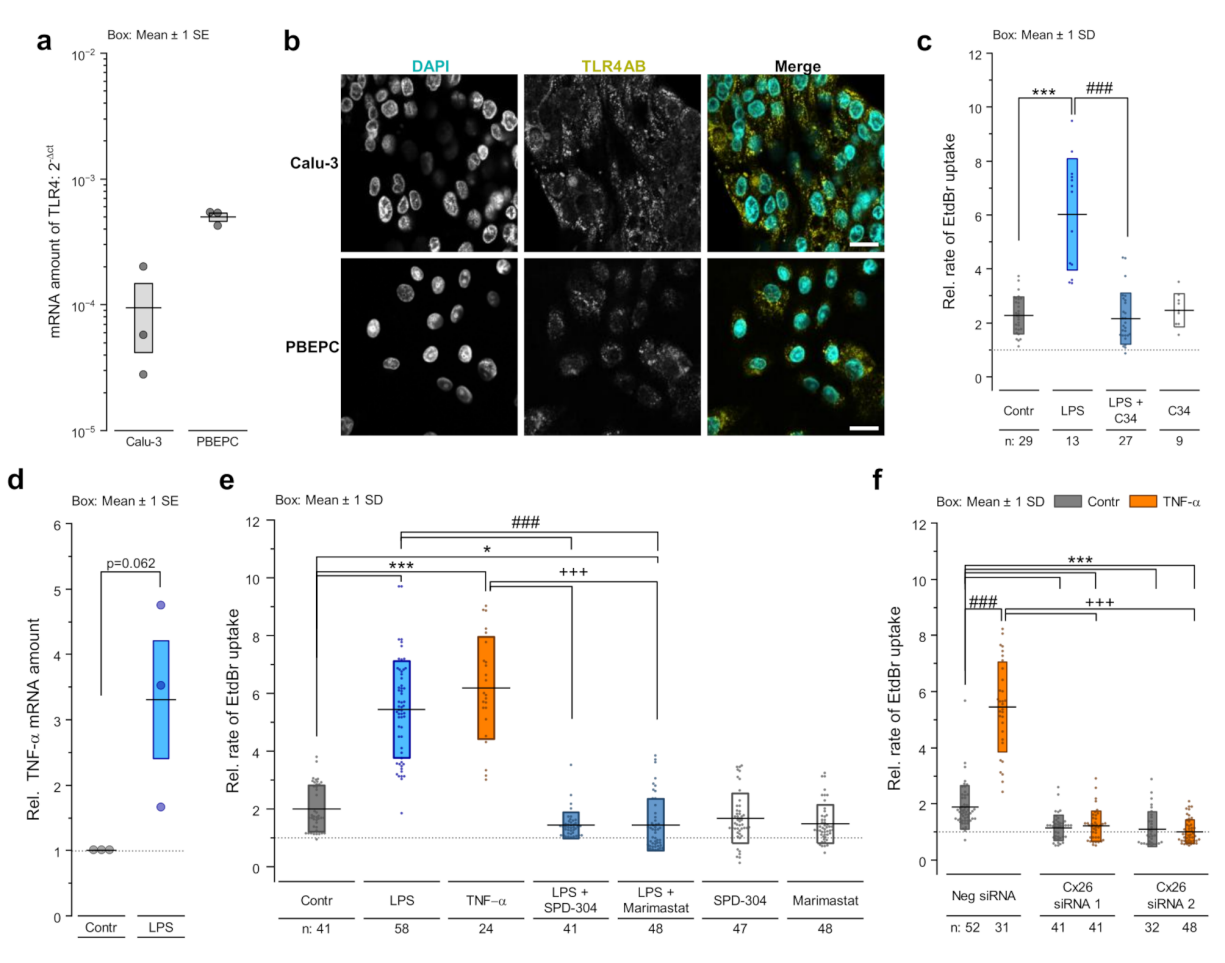
Involvement of TLR4 and TNF-α signaling in the LPS-induced enhancement of Cx26 hemichannel activity. **a** Real-time qRT-PCR experiments for TLR4 mRNA expression in Calu-3 cells and PBEPCs (*n* = 3 biological replicates or donors, respectively). **b** Immunofluorescence staining against TLR4 (yellow) in Calu-3 cells and PBEPCs. Scale bar = 20 μm. **c** EtdBr dye uptake rates in absence of [Ca^2+^]_ex_ relative to the rates obtained in presence of [Ca^2+^]_ex_ in Calu-3 cells treated for 24 h with vehicle, 1 ng/mL LPS ± preincubation (0.5 h) with 20 µM C34 or C34 alone (n = cell patches). Kruskal-Wallis test with Dunn’s multiple comparison test (*p* < 0.001 *** vs. vehicle, *p* < 0.001 ### vs. LPS). **d** Real-time qRT-PCR for TNF-α mRNA amount in Calu-3 cells treated for 3 h with 1 µg/mL LPS (*n* = 3). Unpaired two-tailed *S*tudent*’*s *t*-test. **e** EtdBr dye uptake rates in absence of [Ca^2+^]_ex_ relative to the rates obtained in presence of [Ca^2+^]_ex_ in Calu-3 cells treated for 24 h with vehicle, 1 ng/mL LPS ± preincubation (0.5 h) with 10 µM SPD-304 or 10 µM Marimastat, with SPD-304/Marimastat alone or for 1 h with 10 ng/mL TNF-α (n = cell patches). Kruskal-Wallis test with Dunn’s multiple comparison test (*p* < 0.05 *, *p* < 0.001 *** vs. vehicle; *p* < 0.001 ### vs. LPS; *p* < 0.001 +++ vs. TNF-α). **f** EtdBr dye uptake rates in absence of [Ca^2+^]_ex_ relative to the rates obtained in presence of [Ca^2+^]_ex_ in Calu-3 cells in which Cx26 expression was suppressed using respective siRNA and which were treated with 10 ng/mL TNF-α or vehicle for 1 h (n = cell patches). Kruskal-Wallis test with Dunn’s multiple comparison test (*p* < 0.01 **, *p* < 0.001 *** vs. negative siRNA + vehicle; *p* < 0.05 #, *p* < 0.001 ### vs. respective siRNA + vehicle; *p* < 0.001 +++ vs. TNF-α)

**Fig. 4:**
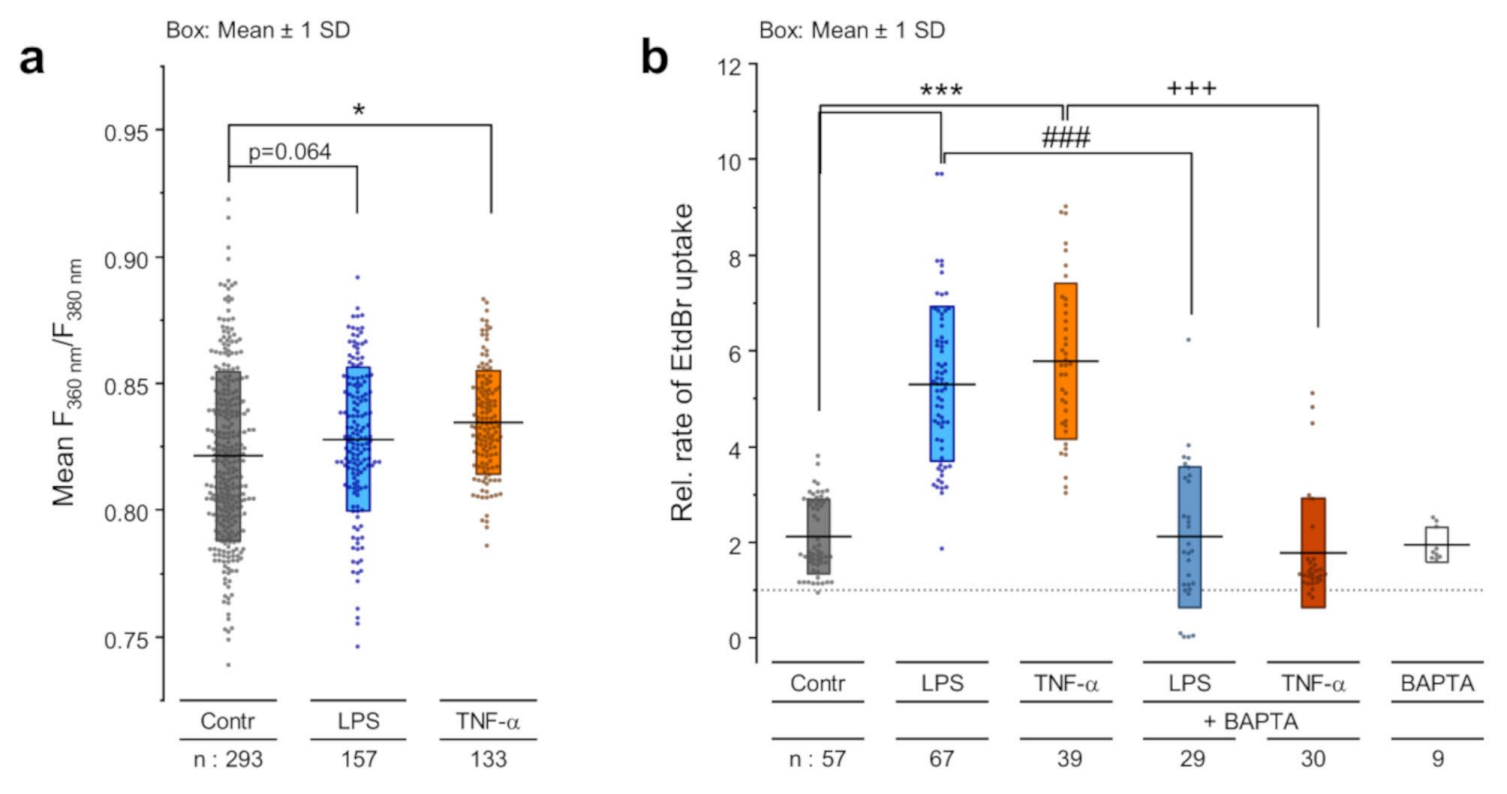
Involvement of intracellular Ca^2+^ in the LPS- and TNF-α-induced enhanced dye uptake rate. **a** Ca^2+^ imaging using Fura-2-loaded Calu-3 cells to estimate baseline [Ca^2+^]_i_ levels (mean F_360 nm_/F_380 nm_ of 5 min measurement) in cells treated with 1 ng/mL LPS for 24 h or 10 ng/mL TNF-α for 1 h (*n* = single cells). One-way ANOVA with Sidak’s multiple comparison test (*p* < 0.05 * vs. vehicle). **b** EtdBr dye uptake rates in absence of [Ca^2+^]_ex_ relative to the rates obtained in presence of [Ca^2+^]_ex_ in Calu-3 cells treated for 24 h with vehicle, 1 ng/mL LPS ± preincubation (0.5 h) with 10 µM BAPTA, with BAPTA alone or for 1 h with 10 ng/mL TNF-α ± preincubation (0.5 h) with 10 µM BAPTA (n = cell patches). Kruskal-Wallis test with Dunn’s multiple comparison test (*p* < 0.001 *** vs. vehicle; *p* < 0.001 ### vs. LPS; *p* < 0.001 +++ vs. TNF-α)

The binding of LPS to its receptor TLR4 is a prerequisite for specific action of LPS on the cells [24, 61]. Real-time qRT-PCR (Fig. 3a) and immunofluorescence staining experiments (Fig. 3b) showed that both PBEPCs and Calu-3 cells express the TLR4 receptor. The suppression of LPS-induced dye uptake by C34, a pharmacological inhibitor of the TLR4 receptor (Fig. 3c, S6), confirms the involvement of TLR4 in the LPS-induced increase in Cx26 hemichannel activity. Furthermore, the release of TNF-α is a well-known cellular response to LPS [62]. Accordingly, we found an increased level of TNF-α mRNA in Calu-3 cells treated with LPS compared to those cultivated under control conditions (Fig. 3d). Moreover, inhibition of TNF-α secretion with Marimastat or prevention of TNF-α binding to its receptor TNFR1 with SPD-304 antagonized the LPS-induced increase in the dye uptake rate in Calu-3 cells (Fig. 3e) suggesting that TNF-α-signaling is required for the LPS-induced enhancement of Cx26 hemichannel activity. We also observed that, similar to LPS, TNF-α itself induced an enhancement of the dye uptake rate in a time- and concentration-dependent manner (Fig. 3e, S7). A significant effect on the dye uptake was first observed after 1 h of exposure to 5 ng/mL TNF-α. The dye uptake rate continuously increased with rising TNF-α concentrations reaching a maximum at 10 ng/mL, twice as high as for control levels. Further increase in the TNF-α concentration up to 20 ng/mL did not correlate with a more enhanced rate of the dye uptake (Fig S7). Despite the differences in kinetics and amplitudes (Fig. S2, S7), the TNF-α-induced increased dye uptake rates were, like for LPS, referable to an enhanced Cx26-hemichannel activity. Likewise, siRNA-mediated repression of Cx26 expression in Calu-3 cells suppressed the enhancement of the dye uptake rate after TNF-α treatment (Fig. 3f). Notably, the TNF-α-induced increase in dye uptake occurred independently of pannexin channels. Indeed, pannexin inhibitors such as low CBX concentrations (100 µM) or spironolactone did not antagonize the effect of TNF-α on the dye uptake in Calu-3 cells (Fig. S8) [7]. We further analyzed the role of [Ca^2+^]_i_ in the enhancement of the dye uptake rates due to LPS and TNF-α using Calu-3 cells. Ca^2+^ imaging measurements showed that treatment of the cells with LPS or TNF-α significantly increased the resting [Ca^2+^]_i_ concentration in the cells (Fig. 4a). Additionally, quenching of [Ca^2+^]_i_ antagonized the increased rate of dye uptake induced by both LPS and TNF-α in BAPTA pre-loaded Calu-3 cells (Fig. 4b), indicating the involvement of Ca^2+^ signaling in the LPS or TNF-α induced enhancement of Cx26 hemichannel activity.

### LPS impacts the barrier function of epithelial cells of the respiratory airways

**Fig. 5:**
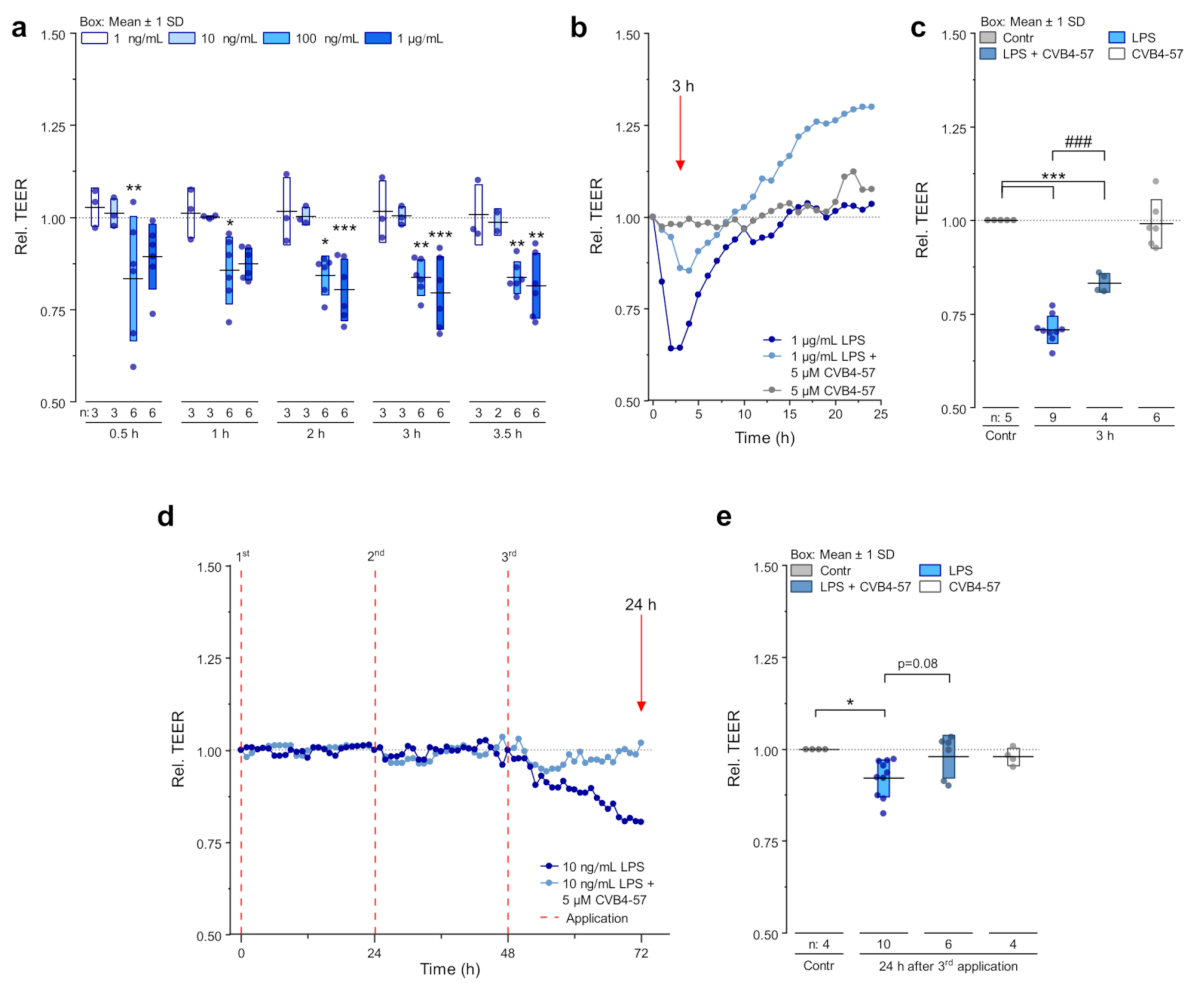
LPS modulates the tight junction barrier in Calu-3 cells, which can be attenuated by CVB4-57. **a** Relative TEER of cells cultivated on transwell inserts and treated for 3.5 h with 1 ng/mL, 10 ng/mL, 100 ng/mL or 1 µg/mL LPS compared to control conditions (n = transwell inserts). One-way ANOVA with Dunnet’s multiple comparison test (*p* < 0.05 *, *p* < 0.01 ** vs. control). **b** Exemplary TEER Measurement over 24 h of cells cultivated on transwell inserts treated with 1 µg/mL LPS ± 5 µM CVB4-57 or CVB4-57 alone. Red arrow: analyzed time point shown in **c**. **c** Relative TEER of cells cultivated on transwell inserts 3 h after treatment with 1 µg/mL ± LPS 5 µM CVB4-57 or CVB4-57 alone (n = transwell inserts). One-way ANOVA with Sidak’s multiple comparison test (*p* < 0.001 *** vs. control; *p* < 0.001 ### vs. LPS). **d** Exemplary TEER measurement of repetitive treatment with 10 ng/mL LPS ± 5 µM CVB4-57 every 24 h for a total time of 72 h (1st, 2nd, 3rd application). Red arrow: analyzed time point shown in **e**. **e** Relative TEER of cells cultivated on transwell inserts 24 h after the 3rd treatment with 1 µg/mL LPS ± 5 µM CVB4-57 or CVB4-57 alone (n = transwell inserts). One-way ANOVA with Sidak’s multiple comparison test (*p* < 0.05 * vs. control)

In parallel to the analysis of Cx hemichannel activity, the effect of LPS on the barrier function of Calu-3 cells cultivated in transwell inserts, which developed a stable TEER of 800–1000 Ωcm^2^, was monitored. Application of high LPS concentrations (≥ 100 ng/mL) induced a continuous reduction of the barrier function as observed by monitoring the TEER (Fig. 5a). After 3 h of treatment, the reduction in TEER reached a maximum of 30% compared to the TEER of control cells. This was followed by a gradual recovery, with TEER returning to the level of untreated cells within 6–8 h. (Fig. 5a-c). Low LPS concentrations (≤ 10 ng/mL) did not affect the TEER when applied once (Fig. 5a). However, repeated application every 24 h resulted in a decreased TEER value of approximately 25% after the third LPS application (Fig. 5d, e). In contrast to the action of high LPS concentrations, the reduction of the TEER by repeated low LPS applications developed slowly (about 24 h). Interestingly, the application of the small molecule CVB4-57 (5 µM), which was able to suppress the enhanced dye uptake in LPS-treated cells (Fig. 1h), also attenuated the reduction in TEER. This attenuation was observed both at high LPS concentrations (Fig. 5b, c) and with repeated low-dose LPS exposure (Fig. 5d, e). For high LPS concentrations, the combined application with CVB4-57 (at 5 µM) reduced the TEER value by approximately 15%, which is significantly less than the 30% reduction induced by LPS application alone (Fig. 5c). Upon repeated application of low LPS concentrations, the reduction of the TEER was almost completely prevented by CVB4-57 at 5 µM (Fig. 5e). Whether the action of CVB4-57 is only limited to cX26 hemichannels is not known, however as already reported [26], CVB4-57 (20 µM) did neither affect the barrier function nor the gap junction coupling of Calu-3 cells.

**Fig. 6:**
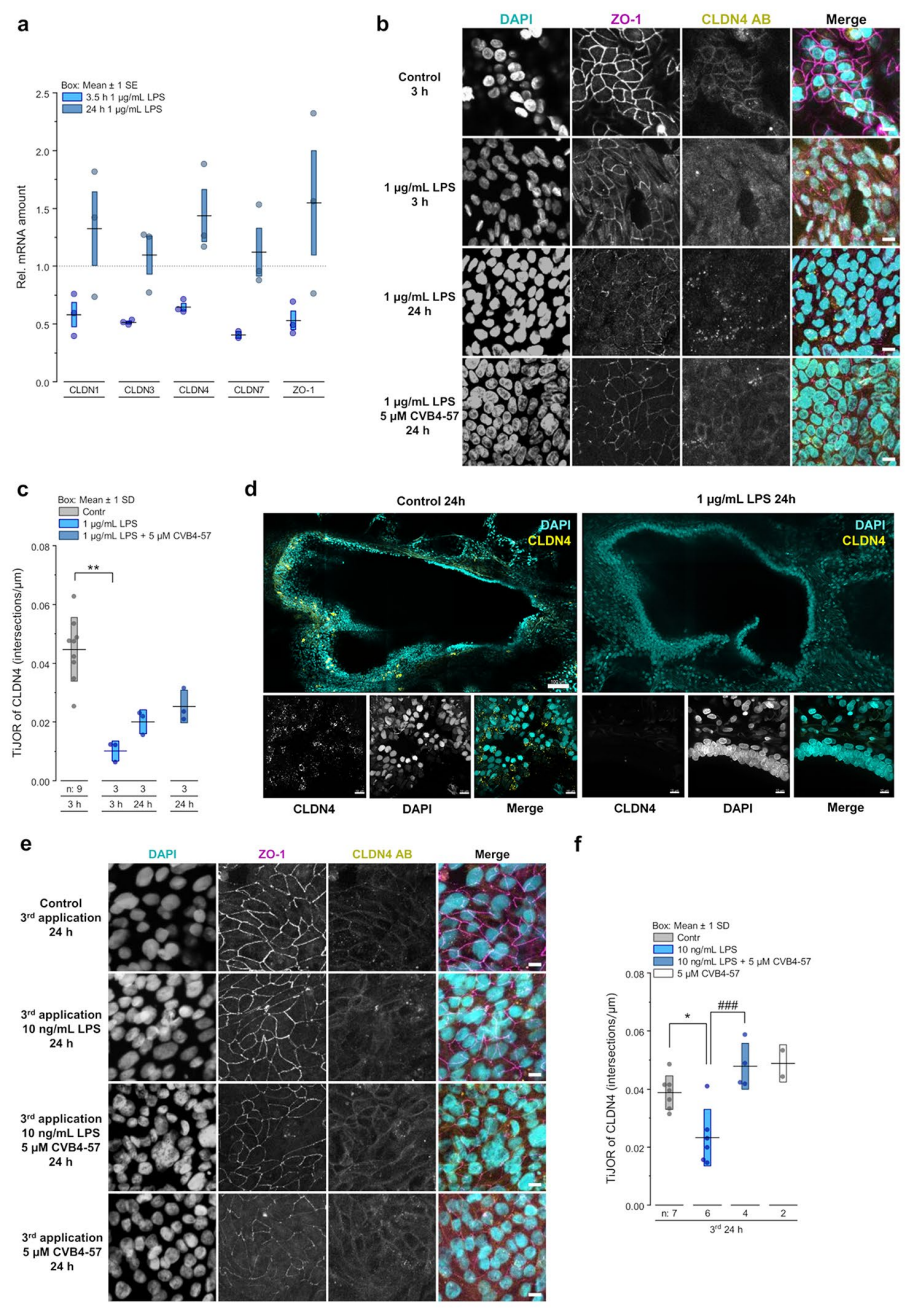
LPS leads to CLDN4 remodeling in epithelial cells of the airways, which can be attenuated by CVB4-57. **a** Real-time qRT-PCR for relative mRNA amounts of CLDN1, CLDN3, CLDN4, CLDN7 and ZO-1 in Calu-3 cells cultivated on transwell inserts after treatment for 3 h and 24 h with 1 µg/mL LPS, respectively (*n* = 3). One-way ANOVA with Dunnet’s multiple comparison test. **b** Exemplary immunofluorescence staining against ZO-1 (magenta) and CLDN4 (yellow) in Calu-3 cells cultivated on transwell inserts treated with 1 µg/mL LPS ± 5 µM CVB4-57 for 3 h and 24 h. Scale bar = 20 μm. **c** Tight junction organization rate (TiJOR, intersections/µm) of CLDN4 after application of 1 µg/mL LPS ± 5 µM CVB4-57 for 3 h or 24 h (n = transwell inserts). Kruskal-Wallis test with Dunn’s multiple comparison test (*p* < 0.01 ** vs. control). **d** Exemplary immunofluorescence staining against CLDN4 (yellow) in human PCLS cultivated for 24 h with 1 µg/mL LPS or culture medium (control). Upper images: 5 × 3 tile scan, scale bar = 200 μm. Lower images: z-stack of 30 μm with one image every 3 μm, scale bar = 10 μm). A similar result was obtained in PCLS of a second donor. **e** Exemplary immunofluorescence staining against ZO-1 (magenta) and CLDN4 (yellow) in Calu-3 cells cultivated on transwell inserts 24 h after 3rd application (application every 24 h) of 10 ng/mL LPS ± 5 µM CVB4-57. Scale bar = 20 μm. **f** TiJOR (intersection/µm) of CLDN4 24 h after the 3rd application of 10 ng/mL LPS ± 5 µM CVB4-57 (n = transwell inserts). One-way ANOVA with Sidak’s multiple comparison test (*p* < 0.05 * vs. control; *p* < 0.001 ### vs. LPS)

We further analyzed whether tight junction proteins such as CLDNs or ZO-1 were affected by LPS treatment and thus involved in the reduction of the TEER. Calu-3 cells mainly express the CLDN isoforms CLDN1, CLDN3, CLDN4 and CLDN7 [15]. Quantitative real-time qRT-PCR showed that the application of a high LPS concentration (1 µg/mL) for 3.5 h decreased the amount of mRNA for the CLDNs and for ZO-1. After 24 h of LPS application, the mRNA levels recovered and even increased compared to cells cultivated under control conditions (Fig. 6a). Immunofluorescence staining against CLDN1, CLDN3 (Fig. S9), CLDN4 and ZO-1 (Fig. 6b, e) showed that the proteins were located at the cell borders. Except for CLDN4, the protein distribution in the tight junctions was not significantly affected by LPS treatment (Fig. S9). For CLDN4, exposure to a high LPS concentration (1 µg/mL) resulted in a reduced fluorescence signal at the cell borders after 3 h of treatment, with the appearance of a nuclear signal observed 24 h post treatment (Fig. 6b). A similar effect was observed after repeated treatment of the cells with a lower LPS concentration of 10 ng/mL (Fig. 6e, S10). Consistent with these observations, we found that the tight junction organization rate (TiJOR) [58] for CLDN4 was reduced by 75% after 3 h of LPS treatment at 1 µg/mL. The recovery of the TEER after 24 h (Fig. 5b) correlated with an incomplete recovery of the TiJOR for CLDN4, which reached approximately 50% compared to control conditions (Fig. 6c). Interestingly, a reduced presence of CLDN4 signal was found in the airways of PCLS treated with 1 µg/mL LPS for 24 h (Fig. 6d). Furthermore, while a single application of low LPS concentrations (10 ng/mL) had no effect on the TEER of Calu-3 cells (Fig. 5d, e), a reduction in CLDN4 TiJOR already occurred 24 h after the first application (Fig. S11) and remained reduced 24 h after the second and third treatments (Fig. S11, 6e, f). As with the LPS-induced alteration of the TEER, CVB4-57, a potential Cx26 hemichannel inhibitor, either partially antagonized the effect of high LPS concentrations on the TiJOR of CLDN4 (Fig. 6c) or completely prevented its reduction after repeated applications of low LPS concentrations (Fig. 6f). These results suggest a potential involvement of Cx26 hemichannel activity in the LPS-induced alteration of the barrier function in Calu-3 cells.

## Discussion

In this report, using human primary bronchial epithelial precursor cells (PBEPCs) expanded from donor lung tissue and Calu-3 cells, we demonstrate that the application of LPS (Fig. 1a, b) and TNF-α (Fig. 3e, f) to epithelial cells of the respiratory airways increases the rate of ethidium bromide (EtdBr) dye uptake into the cells. The EtdBr uptake suggests either a deleterious permeabilization of the cell membrane or an activation of channels large enough to allow EtdBr passage. A detrimental permeabilization of the cell membrane seems unlikely as LPS exposure of to 1 µg/mL for 24 h did not affect the cell viability (Fig. S2c). In terms of channel involvement, Cx hemichannels and pannexin channels could be candidates for the EtdBr uptake [15, 44, 63, 64]. Pharmacological inhibitors of pannexin channels, such as carbenoxolone (CBX) at low concentrations (≤ 100 µM) or spironolactone, did not inhibit the TNF-α-induced reinforcement of the dye uptake (Fig. S6). The enhancement of the dye uptake induced by LPS was inhibited by classical inhibitors of Cx channels such as La^3+^ (Fig. 1a, b). Furthermore, the LPS- and TNF-α-induced increase of the dye uptake rate was reduced in cells in which Cx26 was repressed using specific siRNA (Figs. 1f and 3f). Interestingly, Cx43 siRNA did not suppress the LPS-induced enhancement of the dye uptake (Fig. 1f). The results indicate that the dye uptake and its LPS-induced enhancement was mainly due to the activity of the Cx26 hemichannels. This conclusion is further supported by the observation that the CVB4-57, a diarylheptanoid small molecule known to inhibit the Cx26 hemichannels [25], reduced the enhanced dye uptake rate in LPS-treated cells to control levels (Fig. 1f). The finding of an increased immunostaining signal for Cx26 in epithelial cells of LPS-treated PCLS (Fig. 1e, f) suggests that an increased activity of Cx26 hemichannels may be of pathophysiological significance. Our molecular docking results show that CVB4-57 could bind within two sites of the Cx26 protomers at the interfaces between the NTH and TM1 helices (Fig. 2, S4, S5), where lipid-like molecules may interact with the Cx channel and influence the structural dynamics. For example, in the case of Cx43 hemichannels [33], a derivative of cholesterol and phospholipids, or unresolved lipids (pore lipid sites) [40] were found to stabilize the closed state by binding in this region. Docking experiment studies showed that Cx channel inhibitors such as CBX for Cx46 channels [39] or pilocarpine for Cx43 channels [40] could bind in these regions. A structural study associated with a cross-repositioning of data on Cx26 structures (7QEV) [35] revealed unoccupied cavities in which CVB4-57 could accurately bind (Fig. 2). CVB4-57 may interact with an open Cx26 hemichannel and thereby affect the structural transition to a closed state, finally acting as an inhibitor of the Cx26 hemichannels. Our modelling results indicate that the envelope (linear form) and interactions of CVB4-57 are compatible with putative binding cavities of Cx26. Whether this stabilizes a closed conformation of Cx26 hemichannels remains an open question. Further analyses are necessary to ascertain this and to confirm the predicted binding interactions of CVB4-57 based on our modeling data. This will involve directed mutagenesis, protein isolation and crystallization in the presence of the small molecule, followed by X-ray crystallography and/or cryogenic electron microscopy approaches. While the subject is of great interest, it lies beyond the scope of the present report. For the present, the modelling results support our initial hypothesis that CVB4-57 could bind to Cx26 hemichannels. Furthermore, the modelling identifies the residues that may interact with CVB4-57 in TM1 and TM3 domains (see Fig. 2a, b; Fig. S4). As the TM1 and TM3 domains are frequently cited as delineating the pore of Cx hemichannels [60, 65–67], our modelling is consistent with the current understanding of the mode of function of Cx hemichannels. Consequently, the modelling outcomes suggest potential avenues for the development of small molecules that could be utilised to inhibit Cx hemichannels.

Pharmacological and biochemical experiments showed that LPS affects the Cx26 hemichannel activity via its binding to the TLR4 receptor (Fig. 3a-c, Fig. S6), the classical LPS receptor. Strikingly, the results observed with LPS treatment were completely inhibited by C34, a specific inhibitor of the TLR4 (Fig. 3c, S6). This finding demonstrates a functional link between the inflammatory inducer LPS and Cx26 hemichannels. A similar link between inflammatory inducers and Cx26 hemichannels has already been proposed in the epithelial cells of the epidermis [5]. In the present report, the signaling pathway involving TNF-α appeared to be the functional link between LPS, TLR4 and Cx26 hemichannels. Inhibition of TNF-α secretion or its inactivation thereby preventing binding to its receptor TNFR1, suppressed the LPS-induced enhancement in Cx26 hemichannel activity (Fig. 3e). In addition, TNF-α treatment increased Cx26 hemichannel activity as well as LPS one (Fig. 3e, f). Therefore, we propose a LPS-TLR4-TNF-α-Cx26 chain of functional interactions in the epithelial cells of the respiratory airways. A similar chain of functional interaction of LPS-TLR4-TNF-α and Cx hemichannels has been postulated for different tissues. For example, Cx43 hemichannels were shown to be open in hippocampal astrocytes of LPS-exposed mice offspring. This opening involved TNF-α signaling [68]. Furthermore, similar to our finding of an increased presence of Cx26 in epithelial cells of LPS-treated PCLS (Fig. 1e, f), LPS has been shown to induce *de novo* expression and function of Cx43 and Cx45 hemichannels in myofibers [16].

We observed that the effect of LPS/TNF-α on the Cx26 hemichannel activity in Calu-3 cells correlated with an increase in internal Ca^2+^ concentration [Ca^2+^]_i_ (Fig. 4a). We also found that LPS/TNF-α did not increase the hemichannel activity when applied to cells, in which intracellular Ca^2+^ was chelated after preloading with BAPTA (Fig. 4b). These results indicate the importance of intracellular Ca^2+^ in the LPS/TNF-α-induced enhancement of the Cx26 hemichannel activity. Ca^2+^ is a second messenger, involved in a variety of cellular functions such as gene expression [69], secretion [70, 71] or cytoskeletal reorganization [72]. In our case, we hypothesize that Ca^2+^ was involved in the secretion of TNF-α because the inhibition of TNF-α secretion antagonized the LPS-induced enhancement of the Cx26 hemichannel activity (Fig. 3e). Moreover, we found that quenching internal Ca^2+^ also inhibited the TNF-α-induced enhancement of Cx26 hemichannel activity (Fig. 4b). This suggests that additional Ca^2+^-dependent mechanisms must be involved beyond TNF-α secretion alone. The increased activity of Cx26 hemichannels could also be directly related to the increased [Ca^2+^]_i_. This would be consistent with the observation that elevation of [Ca^2+^]_i_ up to 500 nM increased the activity of Cx hemichannels [7, 73, 74].

In addition to Cx hemichannel activity, we also examined the effect of LPS on the epithelial tight junctions and barrier function. Immunofluorescence staining and subsequent quantitative analysis of the micrographs revealed a remodeling of the CLDNs, especially CLDN4, in response to LPS treatment (Fig. 6a, S9). The remodeling of tight junctions was evident through a reduction in CLDN4 TiJOR, a nuclear localization of CLDN4 immunofluorescence signals in Calu-3 cells (Fig. 6b, c, e, f) and a decreased CLDN4 signal in both the airway epithelium and the sub-epithelium of LPS-treated PCLS (Fig. 6d). The latter finding suggests that this remodeling may be of pathophysiological relevance since intact tight junctions are a prerequisite for the barrier function [75]. We assume that, LPS/TNF-α signaling induced a remodeling of cell-cell contacts in the epithelial cells of the respiratory airways, similar to cardiac tissue [76]. Moreover, the observation of nuclear CLDN4 immunofluorescence staining is consistent with the findings of Schilpp et al. (2021) [28], who reported nuclear localization of CLDN3 in respiratory airway epithelial cells in response to inflammatory stress. However, the function of CLDNs in the nucleus remains unclear. In renal cell carcinoma, nuclear localization of CLDN4 has been associated with epithelial-mesenchymal transition [77], a process known to involve a remodeling of cell-cell contacts [78]. These results suggest that even if the barrier function remains unaffected or is restored after an acute inflammatory response, ongoing changes in the epithelial tissue may occur and persist in the cells after the barrier function has recovered. Presumably, the LPS/TNF-α-induced enhancement of the Cx26 hemichannel activity also involves remodeling of membrane proteins and cell-cell contacts, which may result in the presence of non-apposed hemichannels in the cell membrane. The suggestion is partly supported by the finding of Petecchia et al. 2012 [79] showing disruption of tight junction proteins and membrane disassembly in Calu-3 cells after TNF-α exposure. The remodeling of the tight junctions may, at least in part, explain the effects of LPS on the barrier function observed in our study (Fig. 5). Regarding Cx26, it has been shown that ectopic expression of Cx26 in Calu-3 cells prevents the ouabain-induced downregulation of tight junction barrier and fence functions without the formation of gap junction channels [80]. These observations suggest a functional interaction between a gap junction channel-independent role of Cx26 and the tight junctions. In our results, high LPS concentrations induced a reduction of the TEER by approximately 30% (Fig. 5b, c) and a remodeling of tight junction proteins as evidenced by the strong reduction of CLDN4 TiJOR by 75% (Fig. 6c). The TEER recovered (Fig. 5b), suggesting that the barrier function of the epithelium can recover even from extremely high LPS concentrations. However, we still detected a nuclear fluorescence signal of CLDN4 in immunofluorescence staining and a remained reduced TiJOR of CLDN4 by 30% after TEER recovery (Fig. 6c). While high LPS concentrations (1 µg/mL) are commonly used in pharmacological experiments, they may not be relevant in the context of infection. The low LPS concentrations (10 ng/mL) are more similar to those found in bronchio-alveolar fluid lavage from patients with pneumonia [81, 82]. We observed that low LPS concentrations reduced the TiJOR of CLDN4 to a level that was still observable after TEER recovery in response to high LPS concentrations (Fig. 6c, f). A single application of low LPS concentrations (10 ng/mL) did not affect the barrier function although it was effective in inducing an enhancement of Cx26 hemichannel activity (Fig. 1a, b, S2a, b) and a reduction in the TiJOR of CLDN-4 with simultaneous nuclear signal of CLDN4 (Fig. S11). With repeated applications every 24 h, we observed that low LPS concentrations started to affect the barrier function after the 3rd application (Fig. 5d-f). This suggests that a single infection with an infection-relevant LPS concentration may not significantly impair the barrier function. However, even if the barrier function is not affected, molecular changes such as remodeling of the cell-cell contact region followed by an increased activity of Cx26 hemichannels have occurred and may persist after infection. Such “molecular scars” would make the tissue more vulnerable to further infection. Regarding the role of Cx hemichannels in this mechanism, we demonstrated that CVB4-57 at 5 µM alleviated the reduction of the TEER and TiJOR of CLDN4 induced by 1 µg/mL LPS (Figs. 5b and c and 6b and c). In addition, CVB4-57 completely inhibited the reduction of the TEER as well as the reduction of CLDN4 TiJOR induced by repeated applications of 10 ng/mL LPS (Figs. 5d and e and 6e and f). As our modeling results suggested CVB4-57-Cx26 hemichannel-binding interactions (Fig. 2), CVB4-57 may induce a closure of Cx26 hemichannels in agreement with our recent results [25]. It is likely that the LPS/TNF-α signaling triggers remodeling of the cell-cell contact region. This may affect tight junctions and reinforce the Cx26 hemichannel activity. We propose that the increased Cx26 hemichannel activity in turn participates in the maintenance of the remodeled tight junctions, which then culminates in the alteration of the barrier function of the epithelial system. Working in Cx26-knockout conditions with Cx26-knockout Calu-3 cells would allow further investigation if LPS affects the barrier function and tight junctions of respiratory airway epithelial cells by increasing the activity of Cx26 hemichannels. The generation of Cx26-knockout Calu-3 cells and the subsequent experimental procedures are both very exciting, but far beyond the scope of the present report. Nonetheless, the findings of other researchers that demonstrate the critical role of Cx hemichannel activity in the alteration of the epithelial barrier induced by inflammatory signals [6, 18, 83] provide robust support to our proposition. By reducing the Cx hemichannel activity, as shown by the use of CVB4-57, it may be possible to prevent the remodeling of tight junctions and thereby the downregulation of the barrier function of the epithelium during infection-related inflammation.

## Conclusion

In the present report, we have shown that LPS enhanced the Cx26 hemichannel activity in Calu-3 cells, a model of respiratory airway epithelial cells, as well as in primary bronchial epithelial precursor cells (PBEPCs). This effect was followed by a remodeling of the tight junctions and an alteration of the barrier function of Calu-3 cells. In addition, we have found that LPS significantly upregulated the presence of Cx26 in epithelial cells of human precision cut lung slices (PCLS). Finally, the report has demonstrated that CVB4-57 lowered the Cx26 hemichannel activity and probably prevented the LPS-induced tight junction remodeling by this way, thereby preserving the epithelial barrier function. The report shows that suppression of Cx26 hemichannels may be a good way to pharmacologically protect the barrier function of the respiratory epithelial system during the inflammatory process.

## Electronic supplementary material

Below is the link to the electronic supplementary material.

Supplementary Material 1

Supplementary Material 2

## Data Availability

No datasets were generated or analysed during the current study.
