# Supplementary material for: Repression of Connexin26 hemichannel activity protects the barrier function of respiratory airway epithelial cells against LPS-induced alteration
Source: Cell Commun Signal. 2025 May 16;23:226. doi: 10.1186/s12964-025-02228-6 (PMC12082868; doi:10.1186/s12964-025-02228-6)

Repression of Connexin26 Hemichannel activity protects the Barrier Function of Respiratory Airway Epithelial Cells against LPS-induced Alteration

Tina Lehrich ^1#^, Anne Dierks ^1#^, Masina plenge^1^ Helena Obernolte ^2, 3^, Klaudia Grieger ^2, 3^, Katherina Sewald ^2, 3,^, Frederic Rodriguez ^4, 5^, Lucie Malet ^4,5^, Peter Braubach ^3, 6,^ , Florence Bedos-Belval ^4, 5^, Anaclet Ngezahayo ^1, 7,^ ^✉^

^1^ Institute of Cell Biology and Biophysics, Department of Cell Physiology and Biophysics, Leibniz University Hannover, Hannover, Germany

^2^ Fraunhofer Institute for Toxicology and Experimental Medicine (ITEM) Hannover Department of Area Airway Research, Hannover, Germany

^3^ Biomedical Research in Endstage and Obstructive Lung Disease Hannover (BREATH), German Centre for Lung Research, Hannover Medical School, Hannover, Germany

^4^ Université Paul Sabatier, Toulouse III, UMR 5068, Laboratoire de Synthèse et Physicochimie des Molécules d’Intérêt Biologique, Toulouse, France

^5^ CNRS, UMR 5068, Laboratoire de Synthèse et Physicochimie des Molécules d’Intérêt Biologique, Toulouse, France

^6^ Institute for Pathology, Hannover Medical School, Hannover, Germany

^7^ Center for Systems Neuroscience (ZNS), University of Veterinary Medicine Hannover Foundation, Hanover, Germany

^#^ These authors contributed equally to this work

^✉^corresponding author: ngezahayo@cell.uni-hannover.de; Tel.: +49-511- 762 4568

Keywords:

Connexin channels; Lipopolysaccharide; Airway epithelium; Cytokine; Barrier function; Primary cells, PCLS, Calu-3 cells

**Supplementary Materials**

**Uncropped Western Blot Images used for Fig S3:**

Western Blot 1: 2 biological replicate, 1 technical replicate each

Upper membrane (immunoreaction: Cx26, Cx43, β-Tubulin) loading:

| Lane 1 | 2 | 3 | 4 | 5 | 6 | 7 | 8 | 9 | 10 |
| --- | --- | --- | --- | --- | --- | --- | --- | --- | --- |
| Marker | Negative siRNA | Cx26 siRNA3  + Cx43 siRNA2 | Cx26 siRNA3  + Cx43 siRNA2 | Data irrelevant for this paper | Data irrelevant for this paper | Data irrelevant for this paper | Data irrelevant for this paper | Data irrelevant for this paper | Data irrelevant for this paper |

Lower membrane (immunoreaction: Cx26, Cx43, β-Tubulin) loading:

| Lane 1 | 2 | 3 | 4 | 5 | 6 | 7 | 8 | 9 | 10 |
| --- | --- | --- | --- | --- | --- | --- | --- | --- | --- |
| Negative siRNA | Marker | Cx26 siRNA3  + Cx43 siRNA2 | Cx26 siRNA3  + Cx43 siRNA2 | Data irrelevant for this paper | Data irrelevant for this paper | Data irrelevant for this paper | Data irrelevant for this paper | Data irrelevant for this paper | Data irrelevant for this paper |


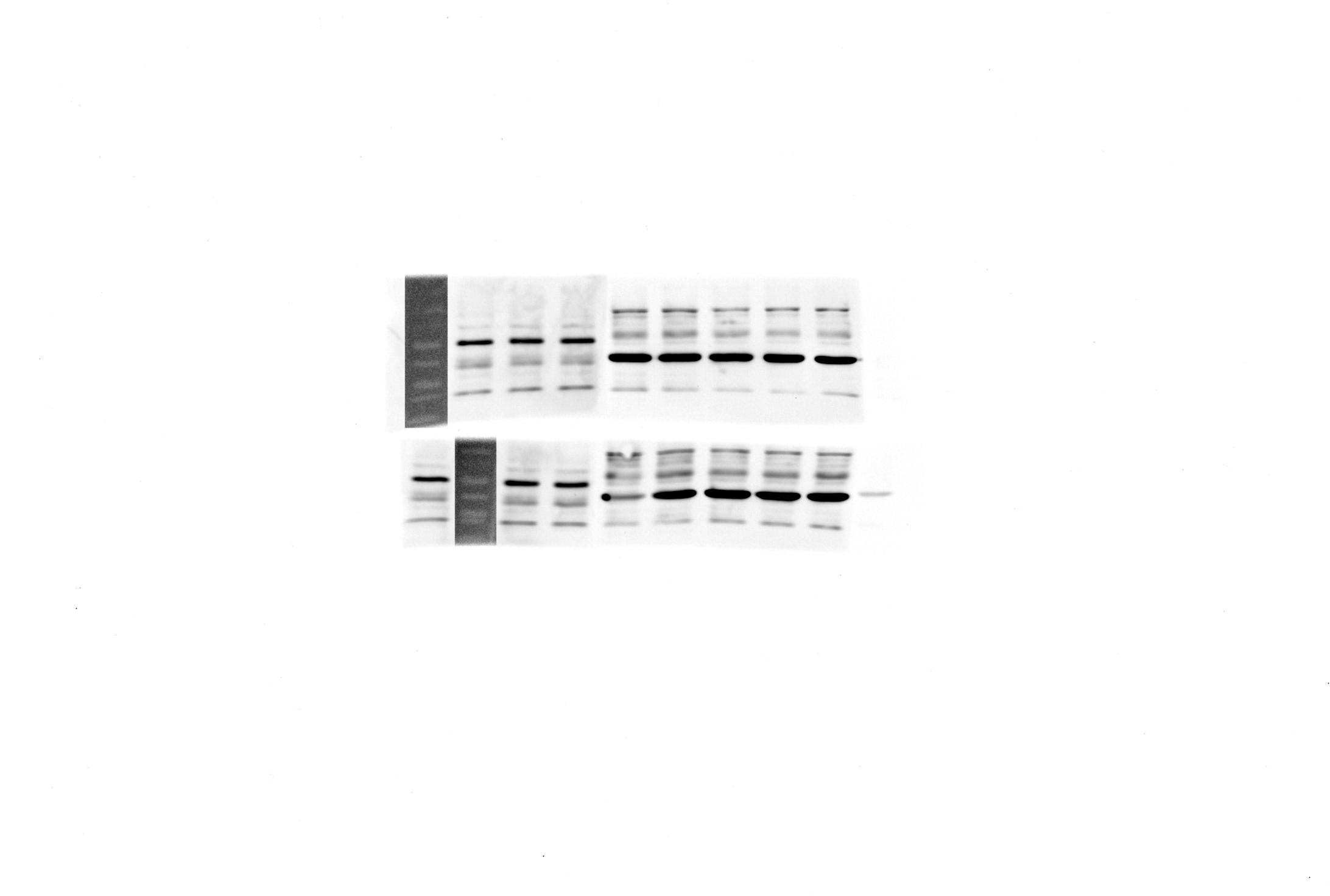


Western Blot 2: 1 biological replicate, 4 technical replicates

Only upper Membrane belongs to respective experiment. Lower membrane shows data irrelevant for this paper. Immunoreaction: Cx26, Cx43, β-Tubulin. Loading:

| Lane 1 | 2 | 3 | 4 | 5 | 6 | 7 | 8 | 9 | 10 |
| --- | --- | --- | --- | --- | --- | --- | --- | --- | --- |
| Marker | Cx26 siRNA3  + Cx43 siRNA2 | Negative siRNA | Cx26 siRNA3  + Cx43 siRNA2 | Negative siRNA | Cx26 siRNA3  + Cx43 siRNA2 | Negative siRNA | Cx26 siRNA3  + Cx43 siRNA2 | Negative siRNA | Marker |


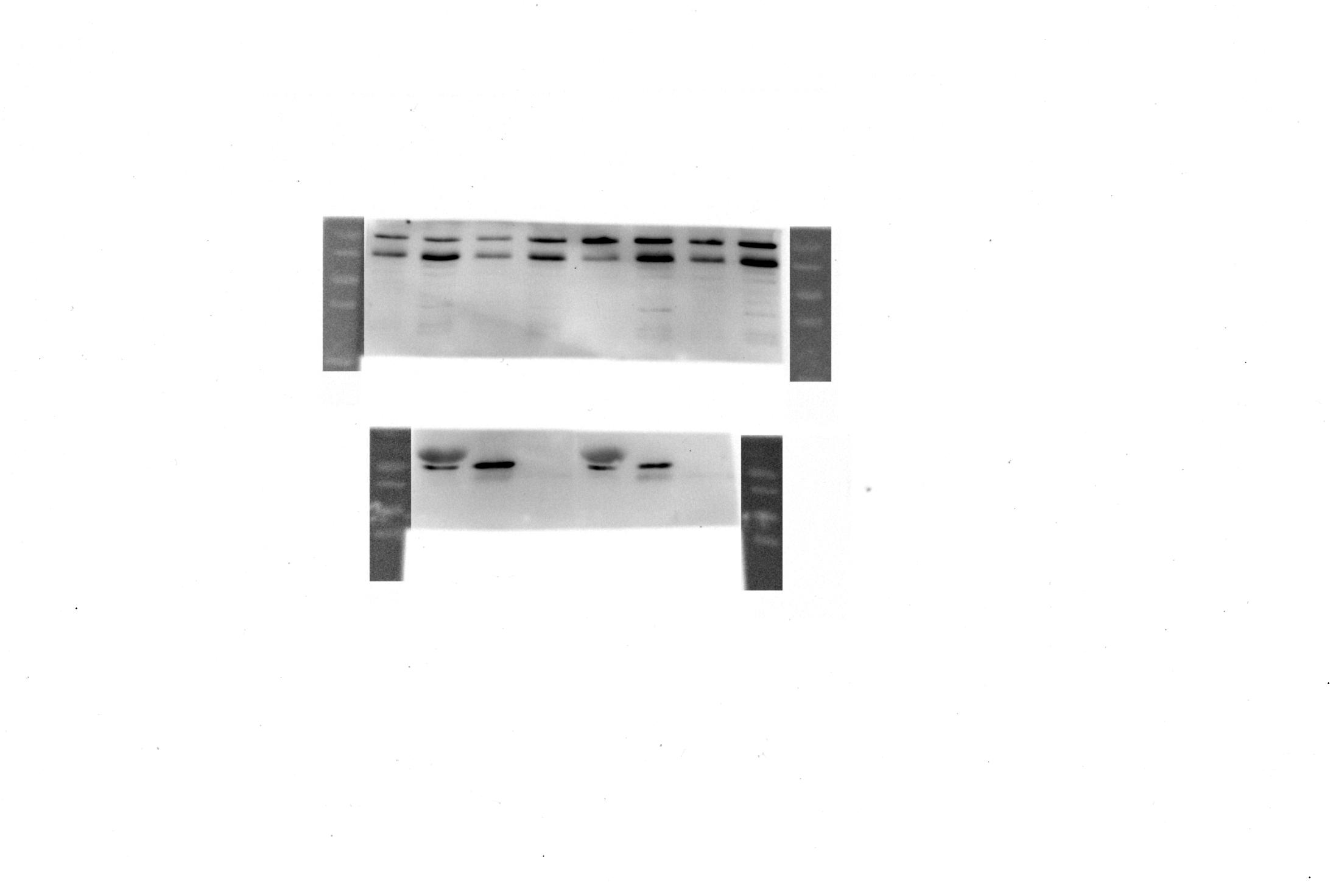

Supplement: Supplementary file 1 — Supplementary Material 1 [file 12964_2025_2228_MOESM1_ESM.docx]
