# Supplementary material for: Repression of Connexin26 hemichannel activity protects the barrier function of respiratory airway epithelial cells against LPS-induced alteration
Source: Cell Commun Signal. 2025 May 16;23:226. doi: 10.1186/s12964-025-02228-6 (PMC12082868; doi:10.1186/s12964-025-02228-6)
Supplement: Supplementary file 2 — Supplementary Material 2 [file 12964_2025_2228_MOESM2_ESM.docx]

Repression of Connexin26 Hemichannel activity protects the Barrier Function of Respiratory Airway Epithelial Cells against LPS-induced Alteration

supplements

Tina Lehrich ^1#^, Anne Dierks ^1#^, Masina Plenge^1^, Helena Obernolte ^2, 3^, Klaudia Grieger ^2, 3^, Katherina Sewald ^2, 3,^, Frederic Rodriguez ^4, 5^, Lucie Malet ^4,5^, Peter Braubach ^3, 6,^ , Florence Bedos-Belval ^4, 5^, Anaclet Ngezahayo ^1, 7,^ ^✉^

^1^ Institute of Cell Biology and Biophysics, Department of Cell Physiology and Biophysics, Leibniz University Hannover, Hannover, Germany

^2^ Fraunhofer Institute for Toxicology and Experimental Medicine (ITEM) Hannover Department of Area Airway Research, Hannover, Germany

^3^ Biomedical Research in Endstage and Obstructive Lung Disease Hannover (BREATH), German Centre for Lung Research, Hannover Medical School, Hannover, Germany

^4^ Université Paul Sabatier, Toulouse III, UMR 5068, Laboratoire de Synthèse et Physicochimie des Molécules d’Intérêt Biologique, Toulouse, France

^5^ CNRS, UMR 5068, Laboratoire de Synthèse et Physicochimie des Molécules d’Intérêt Biologique, Toulouse, France

^6^ Institute for Pathology, Hannover Medical School, Hannover, Germany

^7^ Center for Systems Neuroscience (ZNS), University of Veterinary Medicine Hannover Foundation, Hanover, Germany

^#^ These authors contributed equally to this work

^✉^corresponding author: ngezahayo@cell.uni-hannover.de; Tel.: +49-511- 762 4568

Keywords:

Connexin channels; Lipopolysaccharide; Airway epithelium; Cytokine; Barrier function; Primary cells, PCLS, Calu-3 cells

**Supplementary Figures**

**Fig. S1** **Exemplary time course of a dye uptake experiment.** The fluorescence intensity of ethidium bromide (EtdBr, in AU) in the cells increases after removal of external Ca^2+^ ([Ca^2+^]_ex_). The slope of the linear regression to the dye uptake curve was used to quantify the dye uptake rate, whereas the blue line indicates the slope of the dye uptake when [Ca^2+^]_ex_ was present (2 mM Ca^2+^) and the red line when [Ca^2+^]_ex_ was removed (0 mM Ca^2+^)

​​​​​​

**c**

**b**

**a**

**Fig. S2 Concentration and time dependency of the LPS-induced enhanced dye uptake in Calu-3 cells.** **a** Concentration-dependency of the LPS-induced increased EtdBr dye uptake rate (average relative dye uptake rate ± standard deviation) after application for 24 h compared to control conditions (24 h, 0.2 % H_2_O). Kruskal-Wallis test with Dunn’s multiple comparison test (p < 0.05 *, p < 0.01 ** vs. vehicle). **b**Time-dependency of LPS-induced (1 ng/mL, 100 ng/mL, 1 µg/mL) increased dye uptake rate (average relative dye uptake rate ± standard deviation) compared to control conditions. Kruskal-Wallis test with Dunn’s multiple comparison test (p < 0.05 *, p < 0.01 ** vs. vehicle). **c** Cell viability test (using CASY, OLS^®^) with Calu-3 cells treated with 1 ng/mL, 100 ng/mL or 1 µg/mL LPS for 24 h. (n = analyzed cell samples). One-way ANOVA with Sidak’s multiple comparison test (n. s.: not significant)

​​​​​​​​

**c**

**b**

**a**

**Fig. S3 Validation of siRNA-mediated knockdown of Cx26 and Cx43 in Calu-3 cells. a** Relative mRNA amounts of Cx26 and Cx43 in Calu-3 cells after knockdown with two different specific siRNAs (siRNA1, siRNA2), respectively (n = 3). One-way ANOVA with Sidak’s multiple comparison test (p < 0.01 **, p < 0.01 *** vs. negative siRNA). **b** Exemplary immunoblot against β-tubulin (~ 55 kDa), Cx43 (~ 40 kDa) and Cx26 (~ 25 kDa) for the analysis of Cx isoform knockdown after siRNA transfection. **c** Relative protein amounts of Cx26 and Cx43 in Calu-3 cells (loading control: β-tubulin; vs. negative siRNA) after knockdown with one specific siRNA, respectively (n = 3). One-way ANOVA with Sidak’s multiple comparison test

​​

**a**

​​
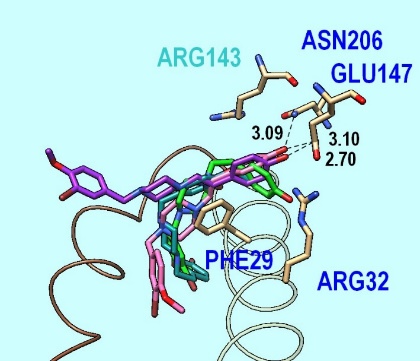


**c**

​​
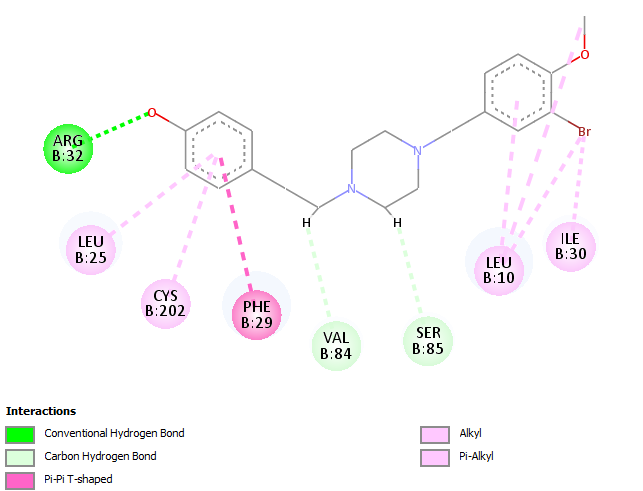

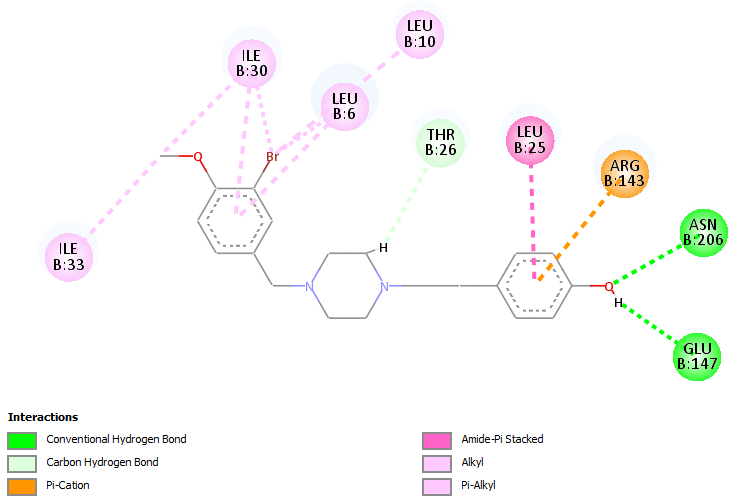


**b**

**Fig. S4: Docking results of best poses and interactions of CVB4-57 for site-01.** **a**Distribution of five best poses based on the crystallographic structure of 6QEV:AB. Three alternative pathways are shown: linear along the cavity (pink pose), constrained (green poses) and towards the cavity between NTHs (purple). The site is shown behind the cavities (brown NTH is in front of the ligands). Atomic distances in Å. **b, c** 2D interaction diagrams of the best pose corresponding to the constrained pathway (**b**, green in **a**) and the linear pathway (**c**, pink in **a**) based on the structure of 6QEV:AB after flexible docking (residues, backbone). For diagram generation Discovery Studio Visualiser (DSV2020) from Dassault Systèmes Biovia (www.3dsbiovia.com) was used.

​​​​

**a**

**b**


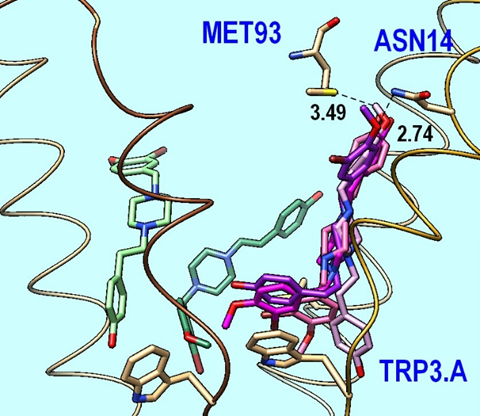

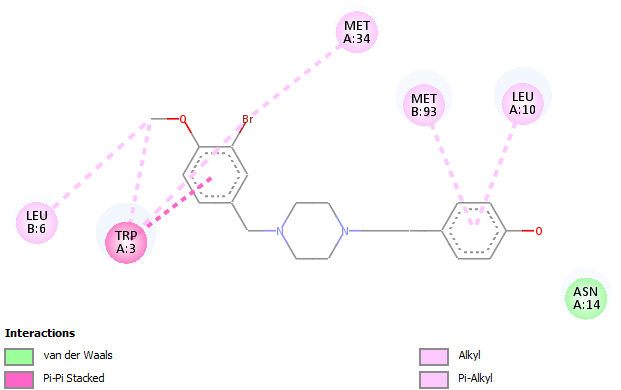


**Fig. S5 Docking results of best poses and interactions of CVB4-57 for site-02. a**Distribution of best poses (pink, green) and fluctuation (levels of pink) for the best pose based on the crystallographic structure of 6QEV:AB. The site is shown from the front of cavity (brown and gold NTHs are in front of the ligands). Atomic distances in Å. **b**2D interaction diagram of the best pose (nb: interaction with ASN14 is not marked but putative) based on the structure of 6QEV:AB after flexible docking (residues, backbone). For diagram generation Discovery Studio Visualiser (DSV2020) from Dassault Systèmes Biovia (www.3dsbiovia.com) was used

**Fig. S6 Involvement of TLR4 in the LPS-induced enhancement of the dye uptake in PBEPCs.** EtdBr dye uptake rates in absence of [Ca^2+^]_ex_ relative to the rates obtained in presence of [Ca^2+^]_ex_ in PBEPCs treated for 24 h with vehicle, 1 ng/mL LPS ± preincubation (0.5 h) with 20 µM C34 or C34 alone (n = single cells). Kruskal-Wallis test with Dunn’s multiple comparison test (p < 0.001 *** vs. vehicle, p < 0.05 # vs. LPS)

​​​​

**b**

**a**

**Fig. S7 Concentration and time dependency of the TNF-α induced enhanced dye uptake in Calu-3 cells. a** Concentration-dependency of TNF-α on the increased EtdBr dye uptake rate (average relative dye uptake rate ± standard deviation) after application for 24 h compared to control conditions (24 h, 0.2 % H_2_O + 0.1% BSA). One-way ANOVA with Dunnet’s multiple comparison test (p < 0.001 *** vs. vehicle). **b** Time-dependency of TNF-α-induced (5 ng/mL, 10 ng/mL) increased dye uptake rate (average relative dye uptake rate ± standard deviation) compared to control conditions. Kruskal-Wallis test with Dunn’s multiple comparison test for 5 ng/mL TNF-α (p < 0.05 *, p < 0.01 ** vs. vehicle). One-way ANOVA with Dunnet’s multiple comparison test for 10 ng/mL TNF-α (p < 0.05 *, p < 0.01 **, p < 0.001 *** vs. control)

**Fig. S8** **The TNF-α induced enhanced dye uptake is independent of pannexin channels.** EtdBr dye uptake rates in absence of [Ca^2+^]_ex_ relative to the rates obtained in presence of [Ca^2+^]_ex_ in Calu-3 cells treated for 1 h with vehicle or 10 ng/mL TNF-α (n = cell patches). Perfusion conditions: 0 mM [Ca^2+^]_ex_, 0 mM [Ca^2+^]_ex_ + 100 µM CBX or + 100 µM spironolactone (Spiro). One-way ANOVA with Sidak’s multiple comparison test (p < 0.001 *** vs. vehicle; p < 0.01 ## vs. TNF-α; n. s.: not significant)

​​​​​​

**d**

**c**

**b**

**a**

​​

**e**

**Fig. S9 High LPS concentrations did not induce major changes on the TiJOR of CLDN1, CLDN3 or ZO-1. a-c** Tight junction organization rate (TiJOR, intersections/µm) of CLDN1 (**a**), CLDN3 (**b**) and ZO-1 (**c**) after application of a high LPS concentration of 1 µg/mL ± 5 µM CVB4-57 for 3 h or 24 h (n = transwell inserts). One-way ANOVA with Sidak’s multiple comparison test (**a, b**) or Kruskal-Wallis test with Dunn’s multiple comparison test (**c**). **d-e**Exemplary immunofluorescence staining against CLDN1 (**d**) or CLDN3 (**e**) (yellow) in Calu-3 cells cultivated on transwell inserts treated with 1 µg/mL LPS ± 5 µM CVB4-57 for 3 h and 24 h. Scale bar = 10 µm. For immunostaining of ZO-1 see Fig. 6

​​​​​​

**d**

**e**

**c**

**b**

**a**

​​**Fig. S10** **Repetitive treatment with low LPS concentrations did not induce major changes on the TiJOR of CLDN1, CLDN3 or ZO-1. a-c** Tight junction organization rate (TiJOR, intersection/µm) of CLDN1 (**a**), CLDN3 (**b**) and ZO-1 (**c**) 24 h after the 3^rd^ application (application every 24 h) of 10 ng/mL LPS ± 5 µM CVB4-57 or CVB4-57 alone (n = transwell inserts). One-way ANOVA with Sidak’s multiple comparison test (**a, b**) or Kruskal-Wallis test with Dunn’s multiple comparison test (**c**). **d-e**Exemplary immunofluorescence staining against CLDN1 (**d**) or CLDN3 (**e**) (yellow) in Calu-3 cells cultivated on transwell inserts 24 h after the 3^rd^ application (application every 24 h) of 10 ng/mL LPS ± 5 µM CVB4-57 or CVB4-57 alone. Scale bar = 10 µm. For immunostaining of ZO-1 see Fig. 6

​​​​

**a**

**b**A

**Fig. S11 Repeated application of low LPS concentrations already induce CLDN4 remodeling 24 h after the first treatment in Calu-3 cells. a**Tight junction organization rate (TiJOR, intersection/µm) of CLDN4 24 h after each of 3 repetitive applications of 10 ng/mL LPS (n = transwell inserts). One-way ANOVA with Tukey’s multiple comparison test (p < 0.05 * vs. control). **b** Exemplary immunofluorescence staining against CLDN4 (yellow) in Calu-3 cells cultivated on transwell inserts 24 h after the each of 3 repetitive applications of 10 ng/mL LPS. Scale bar = 10 µm

**Fig. S12 Duration of siRNA-mediated knockdown of Cx26 in Calu-3 cells.** Relative mRNA amounts of Cx26 in Calu-3 cells after knockdown with specific Cx26 siRNA for 2, 4 and 6 days, respectively (n = 1). Control cells were transfected with negative siRNA
